# Integrated single-cell and bulk transcriptome analysis revealed high plasticity subpopulation and promising diagnosis model for clear cell renal cell carcinoma

**DOI:** 10.1186/s41065-025-00563-9

**Published:** 2025-09-30

**Authors:** Zhongwen Lu, Fanyi Kong, Jiahuan Sun, Jing Ge, Jiajin Wu, Kunpeng Wang

**Affiliations:** 1 Department of Urology, The Affiliated Lianyungang Hospital of Xuzhou Medical University, The First People’s Hospital of Lianyungang, Lianyungang, Jiangsu China; 2 Key Laboratory of Environmental Medicine Engineering, School of Public Health, Ministry of Education of China, Southeast University, Nanjing, China; 3 Department of Urology, First Affiliated Hospital of Nanjing Medical University, No. 300, Guangzhou Street, Nanjing, 210029 Jiangsu Province China

**Keywords:** Single cell and spatial transcriptome, Clear cell renal cell carcinoma, Tumor heterogeneity and plasticity, Machine learning, AXL

## Abstract

Clear cell renal cell carcinoma (ccRCC) is a highly heterogeneous tumor that lacks reliable biological markers for diagnosis and prognostic monitoring. Currently, the differentially expressed genes between paired adjacent normal tissues and ccRCC tumor tissues at single-cell resolution remained to be further discovered. To address this challenge, we performed an integrative analysis of multiple single-cell databases containing paired ccRCC samples. Using the “CopyKAT” algorithm, we accurately identified ccRCC tumor cells. Subsequently, various pseudotime algorithms were employed to identify malignant cells with tumor stem cell-like properties and high plasticity. This cell subgroup exhibited high expression of malignant features, including hypoxia, epithelial-mesenchymal transition (EMT), and proliferation/invasion phenotypes. We then performed differential analysis to identify genes highly expressed in this subgroup and constructed a reliable clinical diagnostic model for ccRCC using multiple machine learning algorithms. Furthermore, we identified AXL as a key gene with significant oncogenic activity, where high expression of AXL correlated with poor patient prognosis. Immune infiltration and spatial transcriptomics analyses further revealed that AXL promotes tumor progression interaction with M2 macrophages. Taken together, our analysis establishes a reliable 13-gene panel diagnostic model and AXL gene as reliable biological markers for ccRCC, providing valuable targets and a theoretical foundation for the development of precision-targeted therapies for ccRCC.

**Supplementary Information:**

The online version contains supplementary material available at 10.1186/s41065-025-00563-9.

## Introduction

Clear cell renal cell carcinoma (ccRCC) is the most prevalent subtype of renal cell carcinoma (RCC), accounting for approximately 70–80% of all RCC cases [1, 2]. According to Cancer Statistics 2024 report, it was estimated that 80,620 new cases of kidney cancer will be diagnosed in the United States this year, with 52,360 cases in men and 28,260 in women [3]. Despite advances in diagnostic modalities and the development of novel targeted therapies, the prognosis for ccRCC remains poor in advanced and metastatic stages, with a 5-year survival rate of less than 10% for patients with distant metastases [4, 5]. However, the heterogeneity of the disease and the lack of effective biomarkers for early detection pose significant challenges [6, 7]. Given the rising global incidence and substantial clinical burden of ccRCC, further research and credible clinical predictors and biomarkers are urgently needed to elucidate the underlying mechanisms of tumorigenesis and to identify novel therapeutic targets for improving survival outcomes.

The tumor immune microenvironment, comprising tumor, immune, and mesenchymal cells, plays a vital role in the success of immunotherapy [8]. Recent advances in single-cell RNA sequencing (scRNA-seq) have significantly enhanced our understanding of the tumor microenvironment (TME) in ccRCC [9, 10]. Through scRNA-seq, distinct subpopulations of cells within the TME have been identified, providing unprecedented insights into their roles in tumor progression, immune evasion, and therapy resistance [11]. ScRNA-seq has revolutionized our understanding of tumor cell plasticity and the role of cancer stem cells (CSCs) in ccRCC [12]. Clear cell renal cell carcinoma is characterized by a high degree of intra-tumoral heterogeneity, driven in part by the remarkable plasticity of tumor cells, which enables them to adapt to diverse environmental pressures, including hypoxia, immune surveillance, and therapeutic interventions [13–15]. ScRNA-seq has provided a powerful tool to uncover the transcriptional and functional diversity of individual tumor cells, revealing distinct cellular states that contribute to tumor progression, metastasis, and resistance to treatment [16]. Moreover, scRNA-seq has elucidated the dynamic transitions between CSCs and non-CSCs, highlighting the plasticity that allows non-CSCs to acquire stem-like properties under stress conditions, such as hypoxia or targeted therapy [17–19]. By dissecting the cellular and molecular landscape of the TME, scRNA-seq offers opportunities for the development of precision medicine strategies aimed at modulating the TME to enhance antitumor immunity and improve therapeutic outcomes for patients with ccRCC.

Machine learning (ML) utilizes a range of powerful algorithms to predict and analyze data, and it is considered the future of biomedical research, personalized medicine, and computer-aided diagnosis [20, 21]. The application of ML models has demonstrated promising accuracy in individual predictions and strong clinical applicability [22]. However, the use of machine learning for the early diagnosis of ccRCC remains underexplored. This study aims to fill this gap by collecting and organizing clinical data from ccRCC patients in the TCGA database, analyzing the influencing factors of key genes, and providing clinical evidence for the precise prediction of tumor initiation and progression in ccRCC. Despite its potential, there is limited explanatory evidence for its clinical application and risk prediction models. To address this, we employed Shapley Additive exPlanations (SHAP) to provide intuitive risk explanations, allowing for more accurate predictions for patients [23, 24]. This tool integrates determinants to generate individual probabilities of clinical events, fulfilling the need for combining biological and clinical models, which ultimately aids in the development of personalized medicine [25]. The aim of this study is to establish reliable biomarkers more suited to ccRCC clinical diagnosis and to develop corresponding predictive models, thereby improving the diagnostic framework for ccRCC and providing clinicians with more valuable insights.

To address above knowledge gap, we conducted a comprehensive and precise analysis of single-cell sequencing data derived from paired ccRCC tumor and adjacent normal tissue samples in this research. Multiple algorithms were utilized to identify and distinguish ccRCC tumor cells. Through various pseudotime analyses and the CytoTRACE cell differentiation model, we characterized the dynamic landscape of tumor cell development. Furthermore, we performed single-cell gene differential expression analysis using several algorithms, integrating machine learning and other methods to comprehensively identify reliable biological biomarkers for the diagnosis and prognostic monitoring of ccRCC in clinical patients. This provides a theoretical foundation for precision treatment and potential therapeutic targets for ccRCC.

## Methods

### Single-cell RNA-seq data acquisition, preprocessing and analysis

Single-cell RNA-seq of paired ccRCC tumor samples and adjacent normal kidney samples were collected from the GEO database, including GSE156632 (Table S1, 10 samples) [26], GSE159115 (Table S2, 8 samples) [19], and GSE178481 (Table S3, 16 samples) [27, 28]. The inclusion criteria for the samples were ccRCC tumor specimens with paired adjacent normal tissue that had not undergone any treatment (ccRCC tumor samples without paired adjacent normal tissue were excluded). The specific clinical, pathological, and patient information is provided in Table S1-S3. Single-cell analysis was primarily performed using the “Omicverse” (Version 1.6.9) and “Scanpy” (Version 1.11.1) Python packages [29, 30]. To obtain high-quality single-cell sequencing results, we first applied the “scCOMPOSITE” algorithm to remove potential doublets from the raw expression matrix [31]. Subsequently, we performed quality control by excluding low-quality cells based on the following criteria: total detected gene count < 500, UMI count < 1000, and mitochondrial gene expression accounting for more than 10% of total gene expression. The remaining single-cell expression matrix was normalized, and 2000 highly variable genes were identified. Batch effects were corrected using the “Harmony” algorithm (theta = 2, lambda = 1, sigma = 0.1, Maximum iteration = 10), and dimensionality reduction and “Leiden” clustering analyses were performed [32]. Cell annotation for subsequent analyses was carried out using published ccRCC-related articles and widely recognized common marker genes [26–28]. Benchmarking test for single-cell integration were performed using “scib” python packages [33]. The evaluation incorporated aggregated scores across key metrics, including preservation of biological variation (Isolated labels, KMeans NMI, KMeans ARI, and cLISI) and the degree of batch effect removal (BRAS, iLISI, KBET, Graph connectivity, and PCR comparation).

## CopyKAT for inferring copy number variations and identifying malignant tumor cells

All epithelial cells were classified as malignant or non-malignant epithelial cells based on the genomic copy number profiles computed from the gene expression matrix using the Bayesian segmentation approach, CopyKAT (Version 1.1.0). CopyKAT could provide inference of genomic copy number and sub-clonal structure of human tumors from sc-RNA data [34]. In this research, we applied the CopyKAT algorithm with default parameters to identify malignant tumor cells among all epithelial cell annotations (minimal number of genes per chromosome for cell filtering = 1, window sizes = 25, segmentation parameter KS.cut = 0.1). For each patient sample, the epithelial cells from the paired adjacent normal tissue were used as reference cells to identify malignant tumor cells in the tumor samples.

## Cell trajectory analysis

To accurately predict cell fate, pseudo-time linkage trajectory analysis was conducted using the “StaVia 2.0” Python package with default parameters applied [35]. For identification of the tumor stem cells (with high plasticity) in ccRCC, we applied computational tool “CytoTRACE 2” to predict cellular potency categories and absolute developmental potential based on single-cell RNA-sequencing data using “Omicverse” Python package, to evaluate and visualize differentiation potential scores of mesenchymal cells across different time points [30, 36].

## Different expression analysis

For discovering differently expressed genes in single-cell data, we utilized several common algorithms, including Wilcoxon rank test (Wilcox), GLM-framework (MAST) and “SEACells” combined with “DESeq2” pseudo-bulk analysis [37, 38]. Genes with false discovery rate (FDR) adjusted *P-*value < 0.05 and the average value of log_2_FC (fold-change) > 1 were considered as differently expressed genes (DEGs). For identification of differentially expressed genes in bulk-RNA sequencing, raw counts format expression matrices between paired tumor and normal tissue samples in TCGA, ICGC and GTEx databases were normalized and calculated using “DESeq2” R package.

## Functional enrichment and pathway annotation

To investigate biological states or function differences of malignant epithelial cells, the Gene Set Enrichment Analysis (GSEA) was used to perform pathway enrichment analysis based on scRNA-seq data. The pathways used in the enrichment analysis, including gene ontology (GO), HallMark, and Kyoto Encyclopedia of Genes and Reactome pathways (www.kegg.jp/kegg/kegg1.html), and the corresponding gene sets were derived mainly from the MsigDB database [39]. For PROGENy functional enrichment and single-cell RNA-seq, we employed “AUCell” and multivariate linear model (MLM) model to infer pathway enrichment scores using “decoupleR” python packages [40, 41] (version 1.8.0).

### Bulk RNA-seq data analysis

We retrieved RNA sequencing data of normal kidney tissues (including both kidney cortex and kidney medulla), adjacent non-tumor kidney tissues, and ccRCC tumor tissues from the TCGA and GTEx databases. To validate the robustness of our findings, we additionally collected data from the ICGC-ccRCC dataset and RNA sequencing data of paired ccRCC tumor tissues and adjacent normal tissues from the GEO databases (GSE36895, GSE40435, and GSE53757). Batch effects were corrected using the “sva” R package (dataset origin was included as covariates in the model), and normalization was performed using log_2_ transformation and standardized. For genes represented by multiple probes, the mean expression value of all probes was taken for further analysis.

## Spatial transcriptomics analysis

The spatial matrices generated after ST data processing from ccRCC tumor samples were analyzed with the Seurat package in R. Low quality spots with minimum detected gene count of 200 genes, genes with fewer than 10 read counts or expressed in fewer than 3 spots were filtered and removed. We performed spatial transcriptomics analysis using “SpaCET” (version 1.3.0), including cell deconvolution [42]. The “SpaCET.deconvolution” function was utilized to decompose the mixture of all spatial transcriptomics (ST) points into malignant cells, immune cells, and stromal cells. Initially, SpaCET estimates the malignant cell fraction using a gene pattern dictionary based on copy number alterations (CNA) and expression changes commonly observed in malignant tumors. Subsequently, immune cell and stromal cell fractions were determined using a constrained regression model, leveraging cellular lineage information derived from single-cell RNA sequencing datasets across various cancer types. Additionally, an unidentified component was included to account for cellular density variations between tissue regions. Signature scoring was performed with the “AddModuleScore” function with default parameters in Seurat. Spatial feature expression plots were generated with the “SpatialFeaturePlot” function in Seurat [43].

## Machine learning for the construction of clinical diagnosis model

We employed three widely used machine learning algorithms to develop a diagnostic model for ccRCC, including LASSO logistic regression, random forest, and XGBoost. Initially, we performed batch effect correction, normalization, and standardization on RNA sequencing data of adjacent normal kidney tissues and ccRCC tumor tissues from the TCGA, GTEx, ICGC, and GEO databases. To ensure the accuracy and robustness of the model, we randomly selected 70% of the samples as the training dataset and reserved the remaining 30% as the validation dataset. Additionally, we utilized SHAP values to visualize the XGBoost model and provide an intuitive representation of the importance score for each variable. For LASSO, we used 10-fold cross-validation via cv.glmnet() to determine the optimal penalty parameter. For XGBoost, we implemented randomized grid search with 10-fold cross-validation to tune hyperparameters including: learning_rate: 0.01–0.3, max_depth: 3–8, subsample and colsample_bytree: 0.6–1.0.

### Clinical samples and immunohistochemistry (IHC) assays

Formalin-fixed, paraffin-embedded (FFPE) tissue of renal clear cell carcinoma and adjacent normal samples were obtained by radical nephrectomy from The Affiliated Lianyungang Hospital of Xuzhou Medical University. Informed consent from all patients was acquired in the study. The study design and protocol were approved by the ethics committee of The Affiliated Lianyungang Hospital of Xuzhou Medical University (KY-20241127001-01). All included patients underwent radical nephrectomy between 2024 and 2025, and clinicopathological data were retrospectively reviewed according to the WHO/ISUP classification and the TNM staging system. All procedures were conducted in accordance with institutional ethical guidelines, and informed consent was obtained from each patient. Tumor tissues and adjacent normal renal tissues were collected and histologically confirmed by two independent board-certified pathologists. Only specimens with unequivocal histopathological diagnosis were included in the study.

For IHC assay, after deparaffinization and rehydration through graded ethanol, antigen retrieval was performed using citrate buffer (pH 6.0) in a microwave oven for 15 min. Endogenous peroxidase activity was blocked by incubation with 3% hydrogen peroxide for 10 min at room temperature. Non-specific binding was minimized by blocking with 5% normal goat serum for 30 min. Sections were then incubated overnight at 4 °C with primary antibodies. The following day, sections were washed and incubated with HRP-conjugated secondary antibodies, followed by chromogenic detection using DAB substrate and counterstaining with hematoxylin. Immunoreactivity was independently evaluated by two experienced pathologists who were blinded to the clinical data. IHC assays were performed using the primary antibody was diluted as follows: anti-AXL (1:250, Proteintech, China).

### Experiment validation

The ccRCC cell lines 786-O (RRID: CVCL_1051), 769-P (RRID: CVCL_1050), Caki-1 (RRID: CVCL_0234) and human renal tubular epithelial cell line (HK-2, RRID: CVCL_0302) were purchased from CellBank China and cultured in RPMI 1640 (786-O, 769-P); McCoy’s 5 A (Caki-1) and DMEM/F12 (HK-2) (Gibco, USA) containing 10% fetal bovine serum and 1% penicillin/streptomycin. Cells were transfected with control siRNA and siRNA-AXL using Lipofectamine 2000 (Invitrogen, USA). To ensure the authenticity and validity of the experimental data, all cell lines were authenticated by short tandem repeat (STR) profiling prior to use. Cell identity was confirmed to match the original ATCC reference profiles. Additionally, routine quality control measures were implemented throughout the study. Mycoplasma contamination testing was performed at regular intervals using PCR-based detection, and all results were consistently negative.

Total RNA was isolated with Trizol reagent (Invitrogen, USA). HiScript III RT SuperMix (Vazyme, China) was used for cDNA synthesis. QRT-PCR assays were performed with SYBR qPCR Master Mix (Vazyme, China) using LightCycler 480 PCR instrument (Roche, Switzerland) according to the manufacturer’s instructions. The primers and siRNA Oligo used are listed in Table S4. Relative mRNA expression level was calculated using the 2 − ΔΔCt method and normalized against GAPDH.

Cell proliferation was measured using the CCK-8 Cell Counting Kit (Vazyme, China). The absorbance was measured at 450 nm with a microplate reader. For the colony formation assay, 786-O cells were seeded into 6-well plates and incubated for 7 days. Colonies were fixed in 4% paraformaldehyde for 20 min, washed with PBS twice, and stained with 0.1% crystal violet for further analysis.

### Tumor cells and macrophage co-culture assay

The human monocytic cell line THP-1 (RRID: CVCL_0006) were seeded at a density of 5 × 10⁵ cells/mL and treated with 100 ng/mL phorbol 12-myristate 13-acetate (PMA) for 48 h. To evaluate tumor-induced macrophage polarization, differentiated THP-1 cells were co-cultured with 786-O human clear cell renal carcinoma cells using a Transwell system (Corning, USA). THP-1 cells were seeded in the lower chamber, and 786-O cells were placed in the upper chamber to allow paracrine interaction without direct cell-cell contact. Co-culture was maintained for 48 h under standard conditions. After co-culture, THP-1 cells were harvested for further phenotypic characterization by quantitative PCR, Western blotting, or flow cytometry analysis of polarization markers, including CD163, CD206 and ARG1 for M2 macrophages.

### Statistical analysis

Bioinformatic analyses were performed using R software (version 4.4.2). Data are presented as mean ± standard deviation. Student’s t-test was used to determine the statistical significance of differences between two groups, while ANOVA was used for multiple group comparisons. Fisher’s exact test, Chi-square test, Wilcoxon signed-rank test and logistic regression analysis were used to assess the association between gene expression level and clinicopathological characteristics. In the analysis of single-cell RNA sequencing data, differences in cell-type proportions between groups were assessed using the Wilcoxon rank-sum test. The diagnostic performance was evaluated using receiver operating characteristic (ROC) curve analysis. For the analysis of single-cell distribution preferences, we employed the Ro/e algorithm. A Ro/e > 1 indicates that the cell subgroup is enriched in the respective sample. For survival analysis, we utilized the Log-Rank test and Kaplan-Meier survival curves to assess patient prognostic indicators, including overall survival (OS), disease-specific survival (DSS), and progression-free interval (PFI).

## Results

### Single-cell RNA-seq illustrated immune microenvironment landscape between ccRCC and adjacent normal kidney samples

**Fig. 1:**
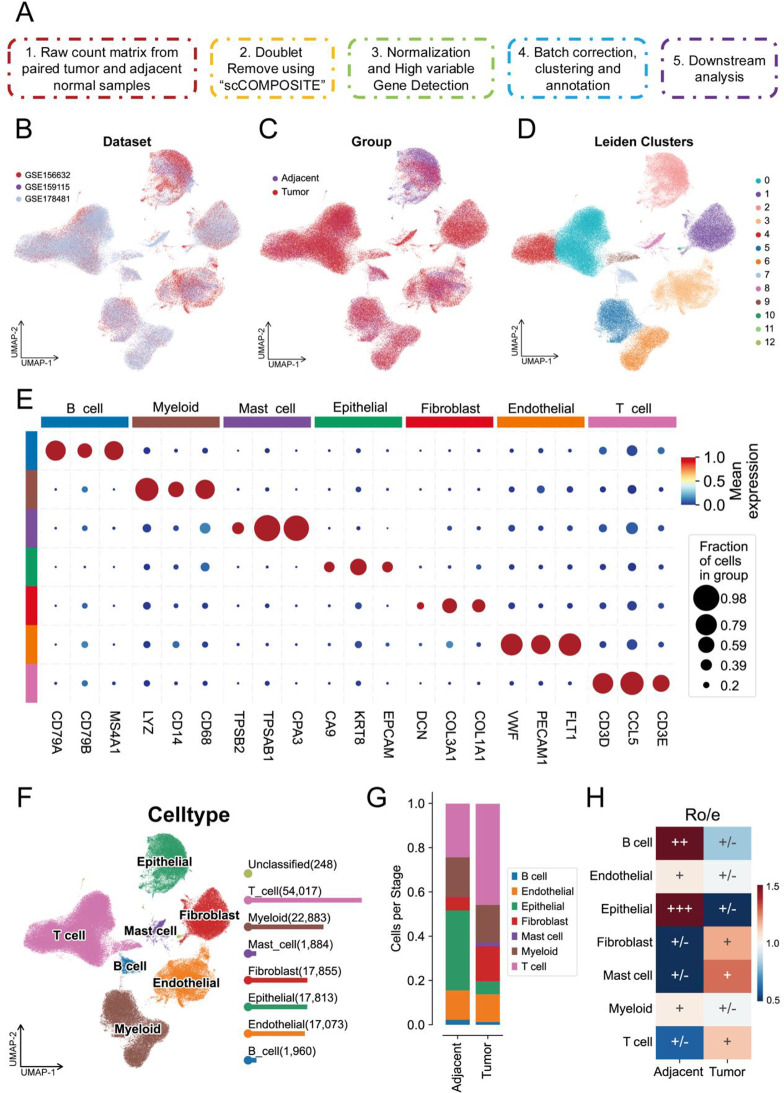
Overview of the scRNA-seq analysis pipeline and results. (**A**) Workflow of the quality control and following scRNA-seq analysis pipeline. (**B**) UMAP plot showing the dataset distribution for these datasets. (**C**) UMAP plot showing the grouping of tumor and adjacent normal samples. (**D**) UMAP plot illustrating Leiden clusters, with distinct colors representing different clusters. (**E**) Dot plot representing the expression of key marker genes across different cell types. The size of each dot indicates the fraction of cells expressing the gene, and the color intensity indicates the mean expression level. (**F**) UMAP plot depicting the distribution of cell types. (**G**) Bar plot showing the number of cells for each cell type in tumor and adjacent tissues. (**H**) Ro/e index illustrating the distribution levels of different cell types across tumor and adjacent normal samples

To obtain high-quality and reliable single-cell atlases of paired samples of clear cell renal cell carcinoma (ccRCC) and adjacent normal kidney tissue, we performed re-quality control and analysis on three single-cell sequencing datasets. The schematic diagram of the main steps is shown in Fig. 1A. In brief, we obtained the raw single-cell expression matrices of paired samples, applied the “scCOMPOSITE” algorithm to remove doublets, and excluded low-quality cells (Figure S1A). Subsequently, the data were normalized, batch effects were removed, cell subpopulations were identified and annotated, and further analyses were conducted (Figure S1E). The cell distribution from the three datasets is shown in Fig. 1B, and the distribution of cells from tumor and adjacent normal tissues is shown in Fig. 1C. Cell subpopulations were identified using the “Leiden” algorithm, which identified a total of 13 subpopulations (Fig. 1D), and cell annotation was performed based on marker genes. As shown in Fig. 1E, we identified that the tumor microenvironment in ccRCC primarily consists of B cells (CD79A, CD79B, MS4A1), myeloid cells (LYZ, CD14, CD68), mast cells (TPSB2, TPSAB1, CPA3), epithelial cells (EPCAM, KRT8, CA9), fibroblasts (DCN, COL3A1, COL1A1), endothelial cells (VWF, PECAM1, FLT1), and T cells (CD3D, CD3E, CCL5). The distribution of cell subpopulations is shown in Fig. 1F and Figure S1C-D. We then conducted an analysis of cell proportions, which revealed a significant increase in T cell proportion in tumor tissue, accompanied by a significant decrease in epithelial cell proportion (Fig. 1G and Figure S1B). Additionally, we performed a Ro/e cell preference analysis, which showed that B cells and epithelial cells were significantly enriched in adjacent normal tissue, while T cells, fibroblasts, and mast cells were significantly enriched in tumor tissue (Fig. 1H). Overall, these results suggest that immune cells are significantly activated during the development of ccRCC, and epithelial cells gradually transform into tumor cells.

### Identification of malignant ccRCC tumor epithelial cells in scRNA-seq cohorts

**Fig. 2:**
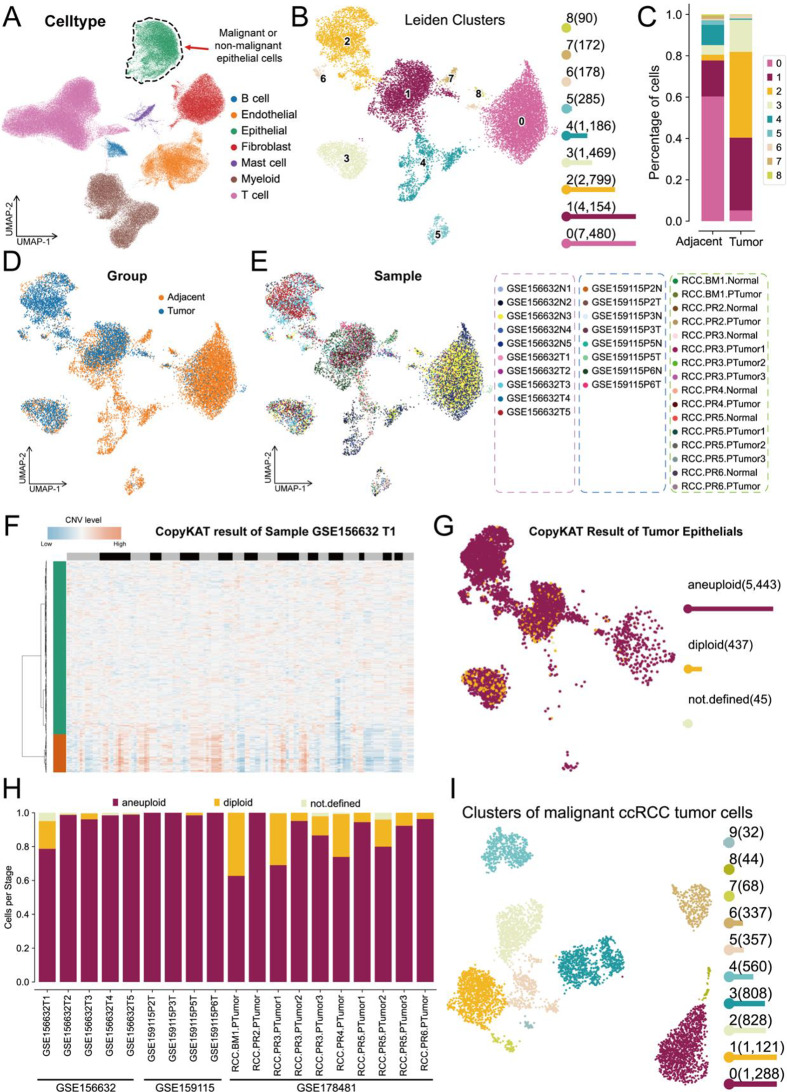
Comprehensive analysis of CNV levels and identification of malignant ccRCC tumor cells. (**A**) UMAP plot showing the distribution of total epithelial cells. (**B**) UMAP plot illustrating the Leiden clusters of total epithelial cells. (**C**) Bar plot showing the percentages of detailed epithelial cell types for each cell type across tumor and adjacent samples. (**D**) UMAP plot of the total epithelial cells grouped by adjacent and tumor samples. (**E**) UMAP plot showing the sample distribution, with different datasets represented by distinct colors. (**F**) Representative heatmap of CopyKAT results for sample GSE156632 T1. (**G**) UMAP plot of CopyKAT predicted results across tumor epithelial cells. (**H**) Bar plot showing the proportion of aneuploid, diploid, and undefined cells across different samples. (**I**) UMAP plot depicting the clustering of malignant ccRCC tumor cells

We then further focused on the evolution of epithelial cells, as shown in Fig. 2A. Epithelial cells mainly consist of malignant clear cell renal cell carcinoma (ccRCC) tumor cells and non-malignant epithelial cells. Therefore, we further subdivided all epithelial cells into subpopulations, as shown in Fig. 2B. All epithelial cells were classified into 9 subpopulations. Subsequent cell proportion analysis revealed that Subpopulation 0 was significantly enriched in normal tissues from adjacent non-tumor samples, while Subpopulations 2 and 3 were significantly enriched in tumor tissues (Fig. 2C). The distribution of epithelial cells from tumor and adjacent normal tissues is shown in Fig. 2D. The cell distribution from different patient tissue samples is shown in Fig. 2E. To further distinguish malignant epithelial tumor cells from all epithelial cells, we performed a copy number variation analysis using the CopyKAT algorithm. As shown in Fig. 2F, taking the first sample from the GSE156632 dataset as an example, using epithelial cells from adjacent normal tissue as a reference, we observed significant copy number amplification and loss events in the epithelial cells of tumor tissue. We then performed CopyKAT analysis on all samples and identified a total of 5443 significantly polyploid cells and 437 haploid cells (which were determined to be normal cells contaminating the tumor samples) (Fig. 2G). The distribution of malignant tumor epithelial cells in each tumor sample from the datasets is shown in Fig. 2H. We then selected malignant epithelial tumor cells from ccRCC for further “Leiden” clustering analysis, which identified 10 distinct cell subpopulations for further analysis (Fig. 2I). Taken together, by employing different algorithms, we precisely identified the expression patterns of malignant tumor cells (epithelial cells) derived from ccRCC patient tumor samples.

### Non-negative matrix factorization and trajectory analysis highlighted the high-plasticity population during ccRCC tumorigenesis

**Fig. 3:**
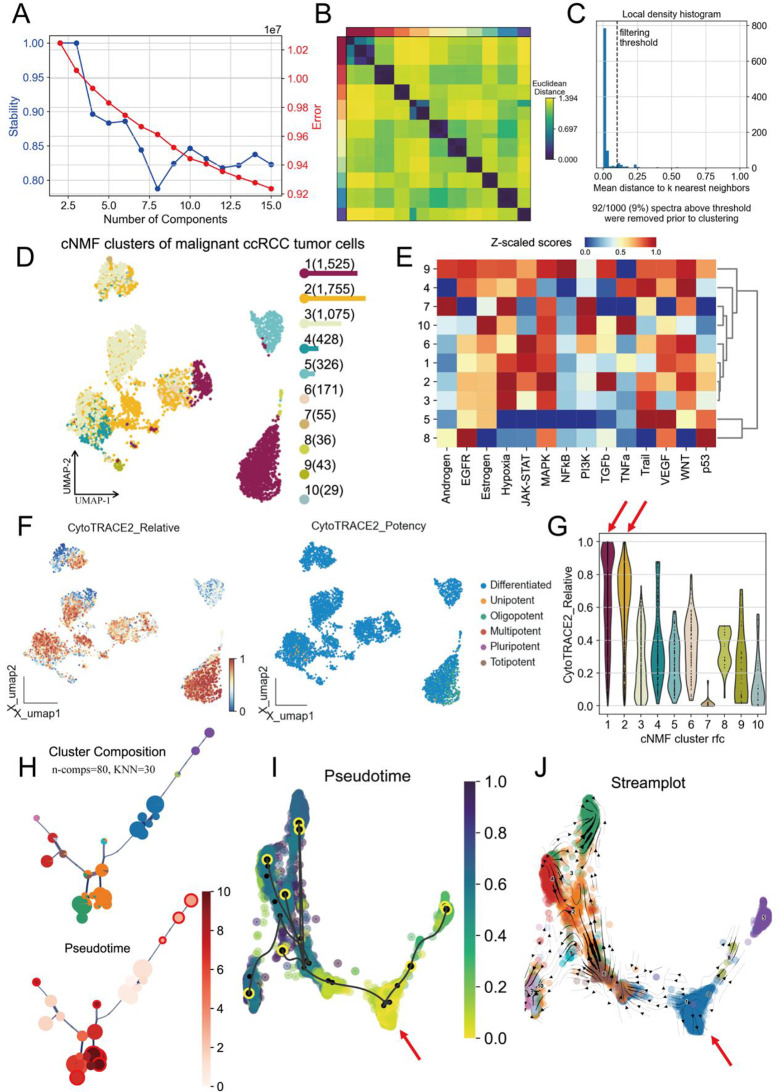
cNMF analysis of malignant ccRCC tumor cells and associated functional characteristics. (**A**) Stability plot showing the relationship between the number of components and the stability score and error for the cNMF clustering analysis of malignant ccRCC tumor cells. (**B**) Heatmap displaying the Euclidean distance matrix between different cells. (**C)** Local density histogram showing the filtering threshold applied to the data. (**D**) UMAP plot of cNMF clusters of malignant ccRCC tumor cells. (**E**) Heatmap of Z-scaled scores for various PROGENy signaling pathways across different clusters. (**F**) UMAP plots showing the relative scores of CytoTRACE2 Relative Score and Potency features. (**G**) Violin plot showing the distribution of CytoTRACE2 Relative scores for each cNMF cluster. (**H**) Cluster composition and pseudo-time analysis. The top panel shows cluster composition across the cNMF clusters. The bottom panel shows pseudo-time. (**I**) Pseudo-time plot showing the progression of tumor cells from early to late stages. (J) Stream plot illustrating the trajectories of the malignant ccRCC tumor cells

Given the significant heterogeneity among malignant epithelial tumor cells, we employed the cNMF (consensus Non-negative Matrix Factorization) method to decompose all epithelial cells. We analyzed the data using 2 to 15 principal components and found that as the number of selected components increased, the stability of the decomposition gradually decreased. The results indicated that when 10 principal components were selected, the stability and error rate of the decomposition reached a balance (Fig. 3A). Therefore, we chose 10 principal components as the number of decompositions for the cNMF method. The Euclidean distances between each component are shown in Fig. 3B, indicating that the components are relatively independent of each other. Next, we filtered the selected principal components based on an average distance threshold of 0.1, with 92 out of 1000 (9%) components falling below the threshold and being excluded from further analysis (Fig. 3C). The distribution of the different cNMF clusters is shown in Fig. 3D. We then performed PROGENy functional enrichment analysis on the 10 identified metaprograms. The results suggested that the 1st and 2nd metaprograms were significantly associated with hypoxia, the JAK-STAT pathway, and tumor-related pathways such as MAPK, TGFb, and WNT, which are closely related to the occurrence and development of ccRCC (Fig. 3E). To further identify the differentiation trajectories in the tumor heterogeneity evolution process, we applied the latest CytoTRACE2 trajectory analysis method to score all malignant tumor cells. The relative CytoTRACE2 scores and differentiation potential classification are shown in Fig. 3F. The results indicated that the relative scores for CytoTRACE2 in the cNMF 1 and 2 metaprograms were significantly elevated, suggesting that these cells are in the early stages of tumorigenesis, with strong differentiation potential and cellular plasticity (Fig. 3G). Furthermore, we performed pseudo-time analysis on the malignant epithelial tumor cells using the VIA algorithm. We selected parameters of n-comps = 80 and KNN = 30 to construct the cell trajectory, which was then arranged in pseudo-time (Fig. 3H). Stream plot and trajectory analysis results indicated that the cells in the cNMF 1 and 2 metaprograms were in the early developmental stages of pseudo-time (Fig. 3I-J). In summary, we have characterized the heterogeneity of malignant epithelial tumor cells and identified tumor stem cell-like subpopulations with strong differentiation potential and plasticity during the early stages of tumorigenesis and development.

### Enrichment analysis confirmed that the high-plasticity subgroup is involved in tumor malignancy-associated pathways

**Fig. 4:**
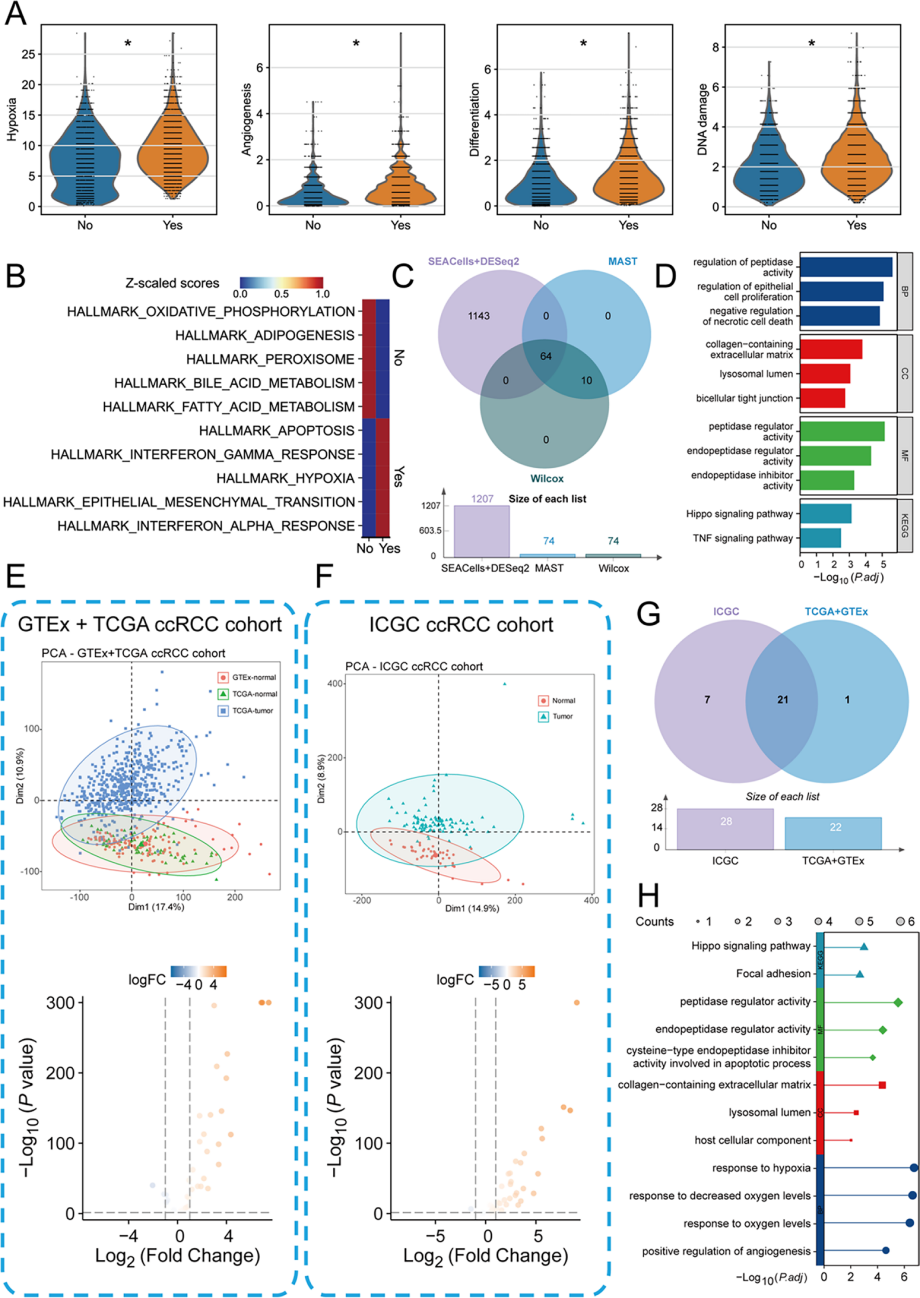
Functional analysis of essential tumor malignancy-associated genes in ccRCC cohorts. (**A**) Violin plots showing the enrichment levels of CancerSEA hypoxia, angiogenesis, differentiation, and DNA damage-related pathways. (**B**) Heatmap of Z-scaled scores for hallmark pathways, including oxidative phosphorylation, adipogenesis, and hypoxia, across ccRCC tumor samples. (**C**) Venn diagram showing the overlap between different methods for identifying significant pathways. (**D**) Bar plot illustrating the top enriched pathways in ccRCC tumor cells. (**E**-**F**) PCA plots for the GTEx + TCGA ccRCC cohort (**E**) and the ICGC ccRCC cohort (**F**). (**G**) Venn diagram illustrating the overlap of significantly differently expressed genes identified in the ICGC ccRCC cohort and the TCGA + GTEx ccRCC cohort. (**H**) Bar plot of significantly enriched pathways across the ICGC and TCGA + GTEx cohorts

To further analyze the biological functions of these highly plastic malignant epithelial tumor cells in the occurrence and progression of ccRCC, we classified all malignant epithelial cells into two states: high-plastic activation (Yes) and non-activation (No) cells. We then performed “CancerSEA” tumor state functional scoring on these subpopulations. The results showed that the highly plastic activated subpopulation exhibited significantly higher scores in hypoxia, angiogenesis, cell differentiation, and DNA damage states compared to the non-activated subpopulation (Fig. 4A). This finding suggests that the activated cell subpopulations play a crucial role in malignant progression and promotion in the development of ccRCC. Additionally, we performed Hallmark pathway enrichment analysis on these subpopulations. The results indicated that the activated subpopulations were significantly enriched in the Hallmark hypoxia pathway, while the non-activated cells were primarily enriched in pathways related to oxidative phosphorylation and other relevant pathways (Fig. 4B). To further characterize and identify the key genes driving the functions of these subpopulations, we utilized “SEACells” combined with “DESeq2” pseudo-bulk analysis (Figure S2A-B), “MAST” algorithm, and Wilcox test to identify marker genes that were highly expressed in the activated state. As shown in Fig. 4C, we identified a total of 64 reliable high-expression genes. GO and KEGG functional enrichment analysis suggested that these genes mainly regulate epithelial cell proliferation and other pathways related to malignant tumor progression (Fig. 4D). Furthermore, to identify more reliable clinical biomarkers, we integrated data from the TCGA-ccRCC, GTEx, and ICGC-ccRCC databases, removing batch effects. We then selected genes that were specifically highly expressed in tumor tissues (Fig. 4E-F). As shown in the Venn diagram in Fig. 4G, we identified 21 marker genes that were highly expressed in ccRCC tumor tissues. These 21 marker genes primarily contributed to the regulation of pathways such as the Hippo signaling pathway (Fig. 4H). In conclusion, by integrating multiple single-cell sequencing datasets and bulk RNA-seq data, we comprehensively identified 21 marker genes with high plasticity that play crucial roles in ccRCC tumor tissues. These genes are proposed as reliable biomarkers for the identification and monitoring of ccRCC occurrence and progression.

### Machine learning developing a reliable 13-gene panel for the diagnosis of ccRCC patients

**Fig. 5:**
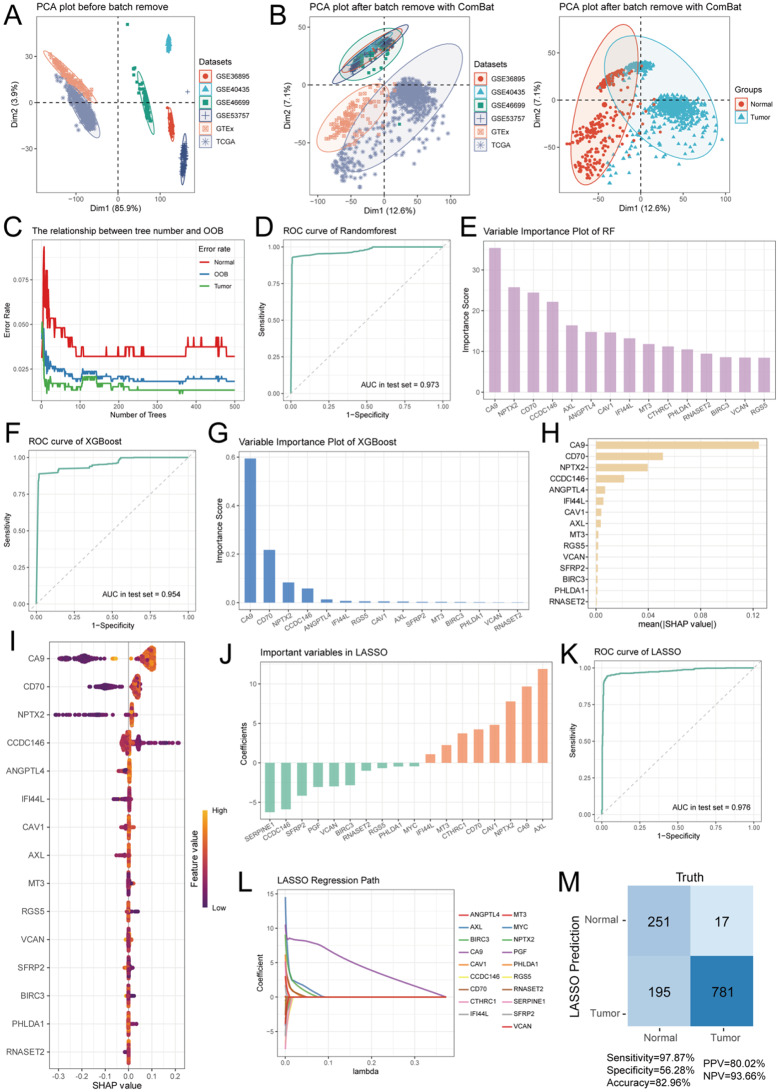
Predictive modeling and variable importance analysis for ccRCC classification. (**A**) PCA plot before batch correction showing the distribution of ccRCC datasets (GSE36895, GSE40435, GSE46699, GSE53757, GTEx, and TCGA). (**B**) PCA plot after batch correction of different datasets and sample types using ComBat algorithm. (**C**) Relationship between the number of trees and out-of-bag error rate for the random forest model. (**D**) ROC curve for the random forest model. (**E**) Variable importance plot for the random forest model. (**F**) ROC curve of the XGBoost model. (**G**) Variable importance plot for the XGBoost model. (**H**) SHAP values for the XGBoost model, visualizing the relationship between feature values and their impact on model predictions for variables. (**I**) SHAP values and feature impact analysis for key variables in the prediction of ccRCC. (**J**) Important variables identified by LASSO regression. (**K**) ROC curve for the LASSO regression model. (**L**) LASSO regression path showing the change in coefficients for selected variables. (**M**) Confusion matrix of the LASSO model’s prediction results for ccRCC classification

To better select reliable biological markers for the clinical differential diagnosis of ccRCC, we further integrated ccRCC bulk RNA sequencing data from multiple paired samples across TCGA-ccRCC, GTEx, ICGC, and GEO for further screening. First, we performed principal component analysis (PCA) on all bulk RNA datasets, which revealed significant batch effects between different datasets (Fig. 5A), potentially introducing noise and interference into the results. Therefore, we initially applied a Log_2_(TPM + 1) transformation to all data and used the Combat algorithm to correct for batch effects between datasets (Table 1). As shown in Fig. 5B, the batch effects between different datasets were significantly eliminated, while the biological signal differences between tumor samples and adjacent normal tissues were retained. Next, we used the random forest algorithm to select marker genes from these 21 candidate genes. By constructing random forest trees, we observed that with an increasing number of trees, the errors between tumor and normal samples, as well as out-of-bag (OOB) samples, gradually decreased and balanced out (Fig. 5C). The ROC curve indicated that the random forest algorithm achieved an AUC value of 0.973 for ccRCC diagnosis (Fig. 5D). Furthermore, we ranked the importance of all variables based on the random forest algorithm and found that CA9, NPTX2, CD70, CCDC146, and AXL were the top five most important marker genes (Fig. 5E). We then applied the XGBoost machine learning algorithm, and the ROC curve showed that the XGBoost algorithm achieved an AUC value of 0.954 for ccRCC diagnosis (Fig. 5F). Based on the importance scores from XGBoost, we identified CA9, CD70, NPTX2, CCDC146, and ANGPTL4 as the top five important marker genes (Fig. 5G). Additionally, we conducted an explainability analysis of the XGBoost algorithm using SHAP values to understand how the models function and evaluate the influence of each factor on ccRCC forecasting. The importance ranking based on average SHAP values also indicated that CA9, CD70, NPTX2, CCDC146, and ANGPTL4 were the top five marker genes (Fig. 5H). The SHAP-based beeswarm plot further confirmed the critical role of these five genes in the diagnostic model (Fig. 5I). The SHAP force plots demonstrate that the bold numbers reflected likelihood predictions [f(x)], while the basic numbers show forecasts made without any input from the designs. For CA9, CD70 and NPTX2, when the feature value is high, its SHAP value trends towards positive, indicating that these genes are positively correlated with the model’s prediction (Figure S3A). For instance, we randomly selected a typical kidney normal sample, and found that based on these genes’ expression level (CA9: -0.197 SHAP value, NPTX2: -0.145 SHAP value and CD70: -0.0855 SHAP value), we conclude that it is a normal renal tissue, which was consistent with the actual result (Figure S3B). Finally, we used LASSO regression analysis to select key genes from the 21 markers. Based on the coefficients, we found that AXL, CA9, NPTX2, CAV1, and CD70 played important roles in LASSO regression. The ROC curve indicated that the LASSO regression algorithm achieved the best AUC value of 0.976 for ccRCC diagnosis among the three machine learning algorithms. We further validated the results using the GEO database and performed confusion matrix analysis, which demonstrated that the LASSO regression algorithm exhibited excellent predictive performance for ccRCC samples. The Venn diagram illustrates that three machine learning algorithms collectively identified 13 essential marker genes of the high-plasticity subgroup for the diagnosis of ccRCC (Figure S3C). In summary, we developing a reliable 13-gene panel and identified the top marker genes among the 21 candidate genes using three mainstream machine learning algorithms, which hold significant clinical value for the diagnosis of ccRCC tumors.

**Table 1:** The scaled and normalized expression level of each gene among signatures in TCGA-ccRCC cohort. (Z-score normalization of log_2_(TPM + 1) values)

Gene	Scaled expression in Normal (*N* = 446)	Scaled expression in Tumor (*N* = 798)	*P*-Value
CA9	0.2 ± 0.1	0.5 ± 0.1	< 0.001
NPTX2	0.2 ± 0.1	0.5 ± 0.2	< 0.001
CD70	0.2 ± 0.1	0.4 ± 0.1	< 0.001
CCDC146	0.3 ± 0.1	0.4 ± 0.2	< 0.001
AXL	0.4 ± 0.1	0.5 ± 0.1	< 0.001
CAV1	0.4 ± 0.1	0.6 ± 0.1	< 0.001
IFI44L	0.3 ± 0.1	0.4 ± 0.1	< 0.001
MT3	0.2 ± 0.1	0.3 ± 0.2	< 0.001
PHLDA1	0.5 ± 0.1	0.5 ± 0.1	< 0.001
RNASET2	0.3 ± 0.1	0.4 ± 0.1	< 0.001
BIRC3	0.4 ± 0.1	0.5 ± 0.1	< 0.001
VCAN	0.3 ± 0.1	0.5 ± 0.1	< 0.001
RGS5	0.5 ± 0.1	0.7 ± 0.1	< 0.001

### Essential gene AXL contributed to ccRCC tumorigenesis and association with tumor immune micro-environment

**Fig. 6:**
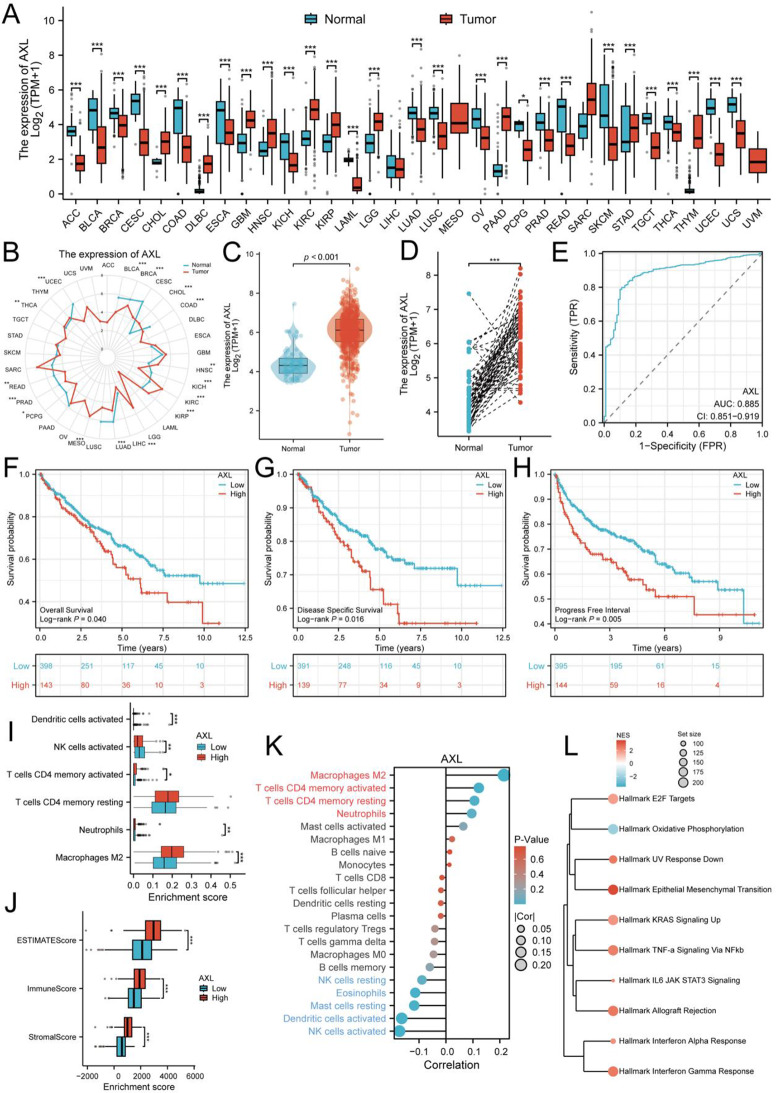
Association of AXL expression with survival and immune features in ccRCC. (**A**) Boxplot showing the expression levels of AXL across various cancer types from the TCGA database. (**B**) Radar plot illustrating the expression of AXL in tumor and normal samples across multiple cancer types. (**C**) Boxplot comparing AXL expression levels between normal and tumor samples in TCGA ccRCC cohort. (**D**) Boxplot showing AXL expression in TCGA ccRCC paired samples. (**E**) ROC curve for AXL expression in TCGA ccRCC dataset. (**F**) Kaplan-Meier survival curves for overall survival in ccRCC patients, stratified by AXL expression. (**G**) Kaplan-Meier survival curves for disease-specific survival in ccRCC patients. (**H**) Kaplan-Meier survival curves for progression-free interval in ccRCC patients. (**I**) Boxplots showing the enrichment scores for immune cell types. (**J**) Boxplots showing the correlation between AXL expression and ESTIMATE, Immune, and Stromal scores. (**K**) Correlation plot showing the relationship between AXL expression and various immune cell types using Cibersoft algorithm. (**L**) Gene set enrichment analysis plot showing significant pathways associated with AXL expression in ccRCC. *: *P* < 0.05; **: *P* < 0.01; ***: *P* < 0.001

Our previous analysis identified key diagnostic biomarkers for clear cell renal cell carcinoma (ccRCC), and found the top 2 essential genes in LASSO regression algorithm are CA9 and AXL. Given the extensive research on CA9 in ccRCC, we focused on the functional role of AXL in this study. First, we analyzed the expression levels of AXL in tumor tissues and normal tissues across various cancers using data from the TCGA and GTEx databases. The results indicated that AXL expression is among the highest in kidney tumor tissues and significantly higher compared to normal tissues or adjacent normal tissues (Fig. 6A). Similarly, we observed the same pattern in the pan-cancer data from TCGA (Fig. 6B and Figure S4A) and paired samples in TCGA (Figure S4B). In the TCGA-ccRCC cohort, the mRNA expression level of AXL was significantly elevated in tumor tissues compared to normal kidney tissues (Fig. 6C). This finding was also observed in paired tumor and adjacent normal tissues from the same patients (Fig. 6D). The diagnostic performance of AXL for ccRCC, assessed via ROC curve analysis, yielded an area under the curve (AUC) of 0.885 (95% confidence interval: 0.851–0.919), suggesting good diagnostic efficacy (Fig. 6E). Next, we evaluated the prognostic predictive performance of AXL in ccRCC patients. High AXL expression was associated with poorer clinical outcomes, including overall survival (OS), disease-specific survival (DSS), and progression-free interval (PFI) (Fig. 6F-H). These results indicate that AXL plays an important role in predicting the prognosis of ccRCC patients. Further analysis focused on the relationship between AXL expression and immune cell infiltration in tumor tissues. Based on the median AXL expression level, we divided all TCGA-ccRCC tumor samples into high- and low-expression groups. Using the ESTIMATE algorithm, we analyzed tumor purity and found that the high-AXL expression group had significantly increased ESTIMATE scores, immune scores, and stromal scores (Fig. 6I). Correlation analysis also showed that AXL mRNA expression levels were positively correlated with ESTIMATE scores, immune scores, and stromal scores (Figure S4C). We then utilized the CIBERSORT deconvolution algorithm to analyze the proportions of different immune cell types in bulk RNA-seq data. A heatmap and boxplot of the distribution revealed that immunosuppressive M2 macrophages, neutrophils, and immunomodulatory CD4 ^+^ T cells were significantly elevated in the high-AXL expression group. Conversely, the levels of tumor-suppressive activated NK cells and antigen-presenting activated dendritic cells were significantly reduced (Fig. 6J and Figure S4D). Correlation analysis also demonstrated that AXL expression was positively correlated with M2 macrophages, CD4 ^+^ T cells, and neutrophils, but negatively correlated with NK cells, mast cells, and dendritic cells (Fig. 6K). Finally, to further investigate the biological functions of AXL in the development and progression of ccRCC, we performed GSEA analysis. The results showed significant enrichment of tumor-associated pathways such as the Hallmark EMT pathway (Fig. 6L and Figure S5A). KEGG analysis also revealed significant associations between AXL and pathways such as the Chemokine Signaling Pathway (Figure S5B). Similarly, Reactome pathway enrichment indicated the involvement of pathways such as Collagen Degradation (Figure S5C). In conclusion, these findings suggest that AXL serves as a reliable biomarker for the diagnosis and prognosis of ccRCC. AXL may contribute to the development and progression of ccRCC by interacting with M2 macrophages and participating in tumor malignancy-associated pathways such as EMT.

### Spatial transcriptome revealed the distribution among ccRCC patients

**Fig. 7:**
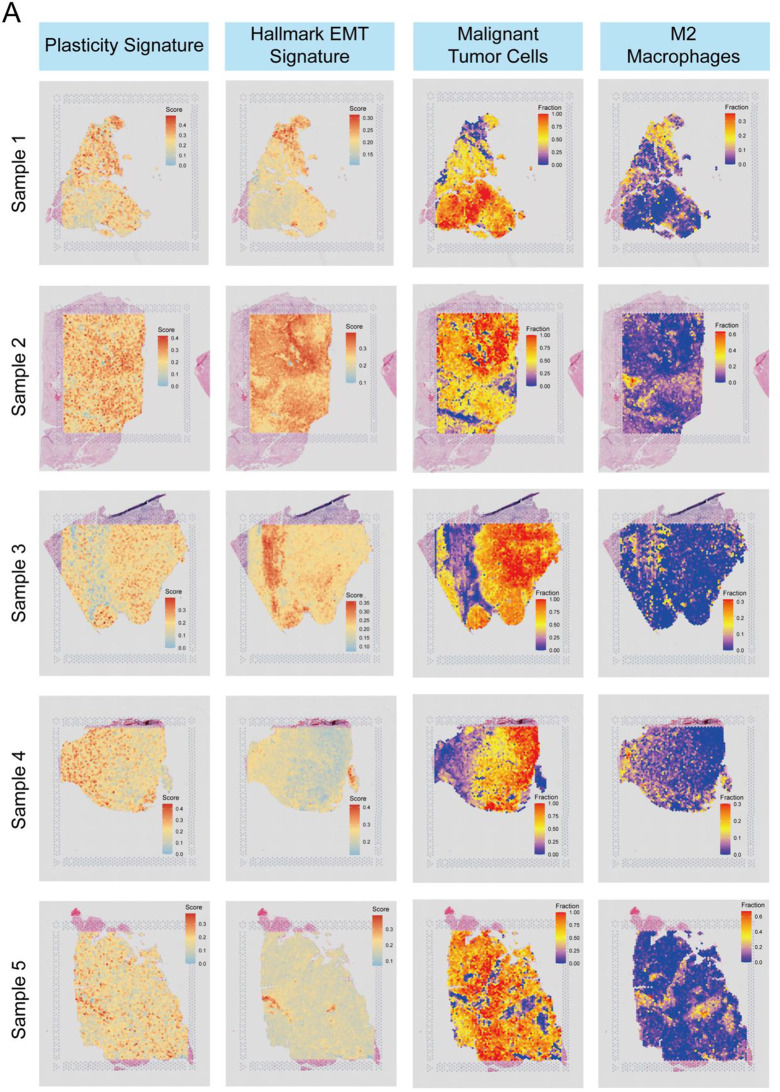
Spatial distribution of molecular signatures and specific cell types in ccRCC tumor samples. (**A**) UMAP plots of five ccRCC tumor samples showing the spatial distribution of key molecular signatures: (1) Plasticity Signature, (2) Hallmark EMT Signature, (3) Malignant Tumor Cells, and (4) M2 Macrophages

Next, we employed spatial transcriptomics sequencing to investigate the spatial distribution of the above results. After rigorous quality control filtering, we found that the spatial distribution of M2 macrophages in tumor samples was closely examined in relation to malignant tumor cells, with notable findings in the proximity between these cell types (Figure S6). In all five tumor samples, M2 macrophages exhibited a distinct pattern, often surrounding the malignant tumor cells, showing a significant spatial proximity (Figure S7). This clustering suggests a potential role of M2 macrophages in the tumor microenvironment, particularly in their interaction with the tumor cells. In addition, the “Plasticity Signature” and “Hallmark EMT Signature” were analyzed across the samples. The “Plasticity Signature” exhibited variable expression within the tumor microenvironment, with stronger associations in the regions corresponding to the malignant tumor cells. The “Hallmark EMT Signature” demonstrated notable spatial enrichment within areas of high tumor cell density, highlighting the potential involvement of epithelial-mesenchymal transition (EMT) processes in tumor progression. The M2 macrophages were notably concentrated around the tumor cell clusters, with these cells showing an increased fraction in areas where tumor cells exhibited higher plasticity and EMT signatures (Fig. 7A). These findings suggest that M2 macrophages may play a crucial role in supporting tumor progression and may be strategically located to influence the tumor microenvironment, particularly in the context of tumor plasticity and EMT. This spatial proximity between M2 macrophages and malignant tumor cells across different samples suggests a potentially significant interaction that warrants further investigation in the context of ccRCC pathology.

#### Experiment validation of essential gene AXL in ccRCC

**Fig. 8:**
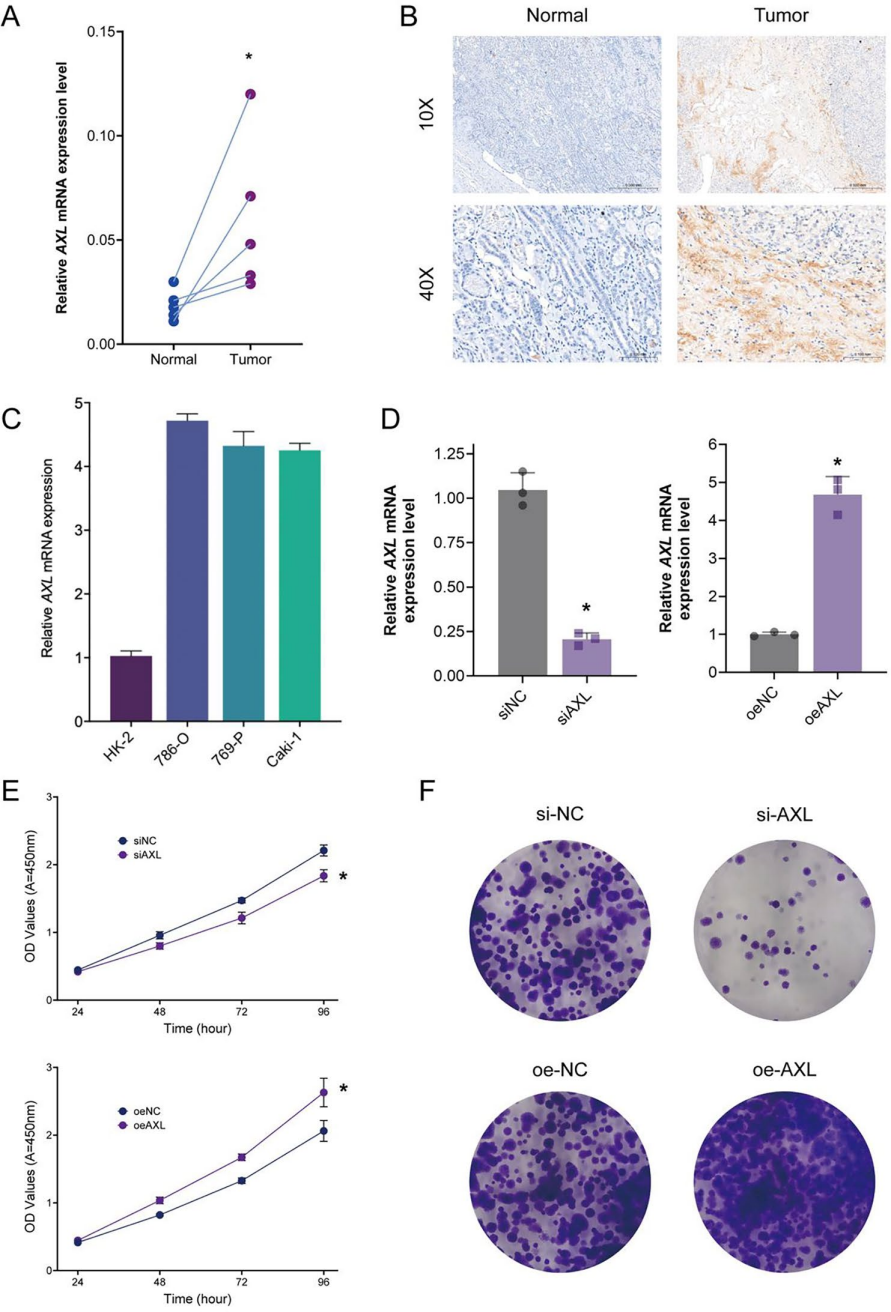
Validation of AXL expression and its impact on ccRCC cell proliferation. (**A**) Quantification of relative AXL mRNA expression levels in ccRCC tumor tissues compared to adjacent normal tissues, as measured by PCR. The results show a significant upregulation of AXL expression in tumor tissues (*n* = 6). *P < 0.05. (**B**) Immunohistochemical staining of AXL in normal and ccRCC tumor tissues at 10X and 40X magnification. (**C**) PCR analysis of AXL mRNA expression in various renal cell lines, including normal renal tubular epithelial cells (HK-2) and ccRCC cell lines (786-O, 769-P, and Caki-1). *P < 0.05. (**D**) PCR analysis confirming the efficiency of AXL knockdown (si-AXL) and overexpression (oe-AXL) in 786-O cells. *P < 0.05. (**E**) CCK-8 assays evaluating the proliferation of 786-O cells with AXL knockdown (si-AXL) and overexpression (oe-AXL). Knockdown of AXL significantly reduces cell proliferation, while overexpression of AXL significantly enhances proliferation. *P < 0.05. (**F**) Colony formation assays showing the effect of AXL knockdown and overexpression on the colony formation ability of 786-O ccRCC cells.

**Fig. 9:**
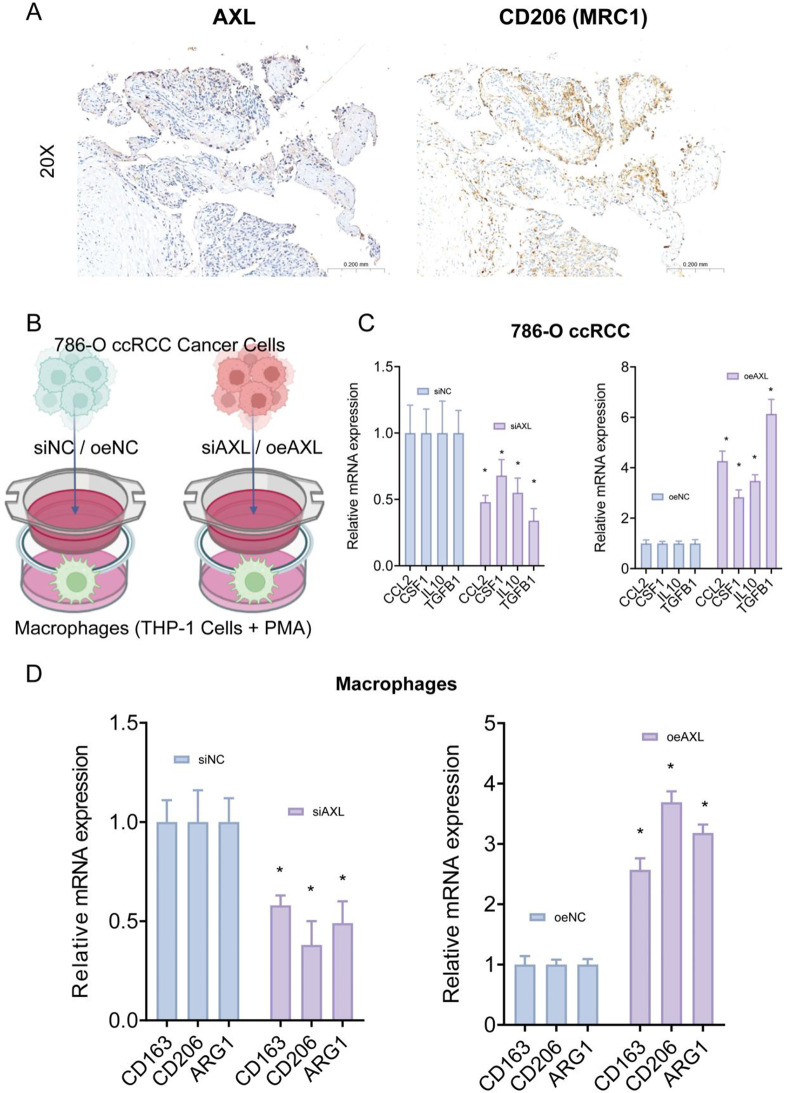
AXL expression in ccRCC tissues and its impact on macrophage polarization. (**A**) Representative immunohistochemical staining of clear cell renal cell carcinoma (ccRCC) tissue sections showing expression of AXL and the M2 macrophage marker CD206 (MRC1). Images were captured at 20× magnification. (**B**) Schematic representation of the transwell co-culture system used to evaluate the impact of AXL modulation in 786-O ccRCC cells on macrophage polarization. (**C**) Quantitative RT-PCR analysis of immunomodulatory cytokines (including CCL2, CSF1, IL-10, and TGF-β) expression level in 786-O cells. (**D**) Quantitative RT-PCR analysis of M2 macrophage markers (CD163, CD206, and ARG1) in macrophages co-cultured with AXL knockdown (si-AXL, left) or AXL overexpression (oe-AXL, right) 786-O cells. Data are presented as mean ± SD (*n* = 3). *P < 0.05

To further validate the biological function of the key gene AXL in the occurrence and development of ccRCC, we first collected tumor tissues from ccRCC patients as well as adjacent normal tissues for PCR and IHC analysis. The results indicated that AXL was significantly overexpressed in ccRCC tumor tissues (Fig. 8A-B). Subsequently, we performed PCR to assess the expression levels of AXL in ccRCC cell lines and normal renal tubular epithelial cell lines. The results showed that AXL expression was markedly elevated in ccRCC cell lines (Fig. 8C). We then constructed AXL knockdown and overexpression 786-O cells and confirmed the efficiency of these constructs via PCR (Fig. 8D). To evaluate the functional significance of AXL, we conducted CCK-8 and colony formation assays, which revealed that AXL knockdown significantly reduced the proliferation of 786-O ccRCC cells, whereas AXL overexpression markedly accelerated cell proliferation (Fig. 8E-F). These results suggest that AXL plays a critical biological role in the occurrence and progression of ccRCC. Additionally, we supplemented the study with immunohistochemistry (IHC) results for AXL and the M2 macrophage marker CD206 in the same ccRCC patient. The results also indicated a spatial colocalization or proximity between AXL and M2 macrophages (Fig. 9A). More importantly, we conducted functional experiments using AXL knockdown or overexpression ccRCC cell lines (786-O) co-cultured with macrophages (Fig. 9B). We found that knockdown of AXL significantly reduced the expression of CCL-2, CSF-1, IL-10, and TGF-β cytokines in ccRCC tumor cells. In contrast, overexpression of AXL led to a marked increase in the expression of these macrophage chemokines. Among them, the increase in TGF-β was the most pronounced. We propose that AXL primarily promotes the polarization of macrophages to the M2 phenotype through the TGF-β signaling pathway (Fig. 9C). We also found that AXL knockdown significantly reduced the expression of the M2 macrophage marker CD206 (MRC1), CD163 and ARG1, indicating that AXL inhibits the differentiation of macrophages into M2-type macrophages, thereby suppressing ccRCC progression. In contrast, overexpression of AXL resulted in a significant increase in the expression of the M2 macrophage marker CD206, promoting ccRCC development (Fig. 9D).

## Discussion

In this study, we conducted a comprehensive multi-omics analysis to explore the dynamic cellular landscape of ccRCC using single-cell RNA sequencing, spatial transcriptomics, and machine learning techniques to identify reliable biomarkers and therapeutic targets. We focused on identifying key tumor cell subpopulations that exhibit high plasticity during tumorigenesis. Clear cell renal cell carcinoma is characterized by its remarkable heterogeneity, and the identification of specific tumor stem cell-like populations has significant implications for understanding its progression and therapeutic resistance. To this end, we employed pseudo-time trajectory analysis combined with advanced algorithms such as “CytoTRACE 2” algorithm to predict the differentiation potential of malignant tumor cells in ccRCC. Our results identified a high-plasticity subgroup of malignant cells, which displayed characteristics reminiscent of tumor stem cells with the ability to transition between distinct cellular states.

By leveraging pseudo-time analysis, we mapped the trajectory of malignant epithelial cells in ccRCC and observed distinct stages of differentiation. This analysis revealed that the early developmental stages, as indicated by the elevated differentiation potential scores, were marked by increased cellular plasticity. These cells were significantly enriched in pathways associated with hypoxia, epithelial-mesenchymal transition (EMT), and other malignant phenotypes known to promote tumor aggressiveness. Such high-plasticity populations are crucial for maintaining the tumor’s ability to adapt to fluctuating microenvironments, thereby supporting tumor progression, metastasis, and therapy resistance. In addition to pseudo-time trajectory analysis, we also employed the “CopyKAT” algorithm to distinguish malignant tumor cells from non-malignant epithelial cells based on copy number variations (CNVs), further corroborating the tumorigenic potential of the identified high-plasticity populations. This dual approach of using pseudo-time and CNV analysis offers a more comprehensive understanding of tumor evolution, enabling the identification of key transition points where tumor cells acquire stem-like properties.

Further exploration through differential expression analysis identified several essential genes that were preferentially expressed in these high-plasticity subpopulations. These findings align with previous reports suggesting that tumor stem-like cells in ccRCC are endowed with the ability to undergo dedifferentiation in response to hypoxia or therapeutic interventions. Our integrative approach, combining pseudo-time analysis with differential expression and functional enrichment, provides new insights into the molecular underpinnings of ccRCC’s heterogeneous.

One of the key findings of our analysis was that we constructed a robust diagnostic model using machine learning algorithms, which integrated multiple datasets from TCGA, GTEx, ICGC, and GEO. By leveraging LASSO logistic regression, random forest, and XGBoost algorithms, we developed a 13-gene panel that demonstrated high accuracy in distinguishing ccRCC from normal kidney tissue, with strong performance in ROC curve analysis. This diagnostic model offers a promising tool for early detection and prognostic prediction, providing a more reliable approach to diagnose ccRCC. These results demonstrated that multiple machine learning algorithms could help interpret the contribution of specific features or biomarkers to clinical diagnosis and prediction. By examining these relationships, researchers can identify critical factors driving predictions, which can aid in understanding disease mechanisms, optimizing diagnostics, or improving therapeutic strategies.

In addition to this reliable 13-gene panel for the diagnosis, we identified that AXL served as a pivotal gene in ccRCC tumorigenesis. High AXL expression was associated with poorer clinical outcomes, including shorter overall survival, disease-specific survival, and progression-free survival, underscoring its potential as a prognostic biomarker. The correlation between AXL expression and immune cell infiltration, particularly its influence on M2 macrophages, further emphasizes its role in immune modulation within the TME. These findings are consistent with recent studies indicating that AXL promotes tumor progression by influencing immune cell dynamics and activating pathways associated with epithelial-mesenchymal transition and metastasis. Furthermore, our spatial transcriptomics analysis revealed that AXL expression is spatially regulated, particularly in regions associated with immune cell infiltration, providing deeper insights and therapeutic targets to ccRCC.

The integration of single-cell RNA sequencing data, spatial transcriptomics, and machine learning algorithms allowed us to uncover the heterogeneity of ccRCC, particularly the plasticity of tumor cells. This approach not only facilitated the identification of tumor stem cell-like populations but also provided insights into the molecular mechanisms underlying ccRCC progression. The identification of key genes, such as AXL, and the development of a machine learning-based diagnostic model mark a significant advancement in ccRCC research, offering potential targets for precision therapy and improving clinical outcomes for patients.

## Conclusions

In conclusion, our study establishes a comprehensive framework for understanding the tumorigenic processes in ccRCC and highlights AXL as a critical biomarker for diagnosis and prognosis. Our results demonstrated the importance of single-cell pseudo-time analysis in unraveling the plasticity of malignant ccRCC cells and identifying tumor stem cell-like subpopulations. These insights not only enhance our understanding of ccRCC biology but also open avenues for the development of targeted therapies aimed at specific tumor cell populations that drive malignancy and therapeutic resistance. The integration of advanced technologies, such as single-cell RNA sequencing and spatial transcriptomics, with machine learning, has the potential to revolutionize ccRCC diagnostics and therapy, providing new avenues for precision medicine in this challenging cancer.

## Supplementary Information

Below is the link to the electronic supplementary material.

Supplementary Material 1

## Data Availability

The datasets generated during the current study are available in the TCGA, ICGC, GTEx and GEO database. All data generated or analyzed during this study were included either in this article Methods section or in the Supplemental Information. Other data that support the findings of this study are available from the corresponding author upon reasonable request. The code used in this research was present in GitHub Repository: https://github.com/wujiajin0920/ccRCC_scRNA.
