# Supplementary material for: Integrated single-cell and bulk transcriptome analysis revealed high plasticity subpopulation and promising diagnosis model for clear cell renal cell carcinoma
Source: Hereditas. 2025 Sep 30;162:198. doi: 10.1186/s41065-025-00563-9 (PMC12487111; doi:10.1186/s41065-025-00563-9)

**Supplementary Materials**

**Supplementary Tables**

**Table S1.** Detailed clinicopathological characteristics of ccRCC patients from single-cell RNA-sequencing in GSE156632 cohort.

**Table S2.** Detailed clinicopathological characteristics of ccRCC patients from single-cell RNA-sequencing in GSE159115 cohort.

**Table S3.** Detailed clinicopathological characteristics of ccRCC patients from single-cell RNA-sequencing in GSE178481 cohort.

**Table S4.** The nucleic acid sequences used in this study.

**Supplementary Figures**

**Figure S1.** Quality control of single-cell data and the distribution of cell composition.

**Figure S2.** Using “SEACells” combined with “DESeq2” pseudo-bulk analysis to identify differentially expressed genes.

**Figure S3.** Machine learning and SHAP values were used to select essential genes and explain the importance of each gene.

**Figure S4.** The expression level of AXL in TCGA pan-cancer datasets and association with immune cell infiltration among tumor micro-environment.

**Figure S5.** Functional enrichment analysis of AXL in TCGA ccRCC dataset.

**Figure S6.** Hematoxylin and Eosin (H&E) staining and quality control results of spatial transcriptomics analysis among 5 ccRCC samples.

**Figure S7.** Spearman correlation analysis to quantitatively assess spatial co-localization.

**Supplementary Tables**

**Table S1.** Detailed clinicopathological characteristics of ccRCC patients from single-cell RNA-sequencing in GSE156632 cohort.

| Patients | GEO number | Tissue type | Gender | Age | Stage |
| --- | --- | --- | --- | --- | --- |
| P1-T | GSM4735364 | ccRCC tumor | Male | 68 | T3 |
| P1-N | GSM4735365 | Adjacent normal | Male | 68 | / |
| P2-T | GSM4735366 | ccRCC tumor | Male | 51 | T2 |
| P2-N | GSM4735367 | Adjacent normal | Male | 51 | / |
| P3-T | GSM4735368 | ccRCC tumor | Male | 68 | T2 |
| P3-N | GSM4735369 | Adjacent normal | Male | 68 | / |
| P4-T | GSM4735370 | ccRCC tumor | Female | 59 | T2 |
| P4-N | GSM4735371 | Adjacent normal | Female | 59 | / |
| P5-T | GSM4735372 | ccRCC tumor | Female | 41 | T2 |
| P5-N | GSM4735373 | Adjacent normal | Female | 41 | / |

**Table S2.** Detailed clinicopathological characteristics of ccRCC patients from single-cell RNA-sequencing in GSE159115 cohort.

| Sample number | GEO number | Tissue type | Patient number | Gender |
| --- | --- | --- | --- | --- |
| SI_18856 | GSM4819726 | ccRCC tumor | P2 | Male |
| SI_18855 | GSM4819727 | Adjacent normal | P2 | Male |
| SI_19704 | GSM4819728 | ccRCC tumor | P3 | Male |
| SI_19703 | GSM4819729 | Adjacent normal | P3 | Male |
| SI_22369 | GSM4819733 | ccRCC tumor | P5 | Male |
| SI_22368 | GSM4819734 | Adjacent normal | P5 | Male |
| SI_22605 | GSM4819735 | ccRCC tumor | P6 | Male |
| SI_22604 | GSM4819736 | Adjacent normal | P6 | Male |

**Table S3.** Detailed clinicopathological characteristics of ccRCC patients from single-cell RNA-sequencing in GSE178481 cohort.

| Sample number | Tissue type | Patient number | Gender | Age | Stage |
| --- | --- | --- | --- | --- | --- |
| RCC-PR2-PTumor | ccRCC tumor | Patient 2 | Male | 50-60 | 3 |
| RCC-PR2-Normal | Adjacent normal | Patient 2 | Male | 50-60 | / |
| RCC-PR3-PTumor1 | ccRCC tumor | Patient 3 | Male | 60-70 | 2 |
| RCC-PR3-PTumor2 | ccRCC tumor | Patient 3 | Male | 60-70 | 2 |
| RCC-PR3-PTumor3 | ccRCC tumor | Patient 3 | Male | 60-70 | 2 |
| RCC-PR3-Normal | Adjacent normal | Patient 3 | Male | 60-70 | / |
| RCC-PR4-PTumor | ccRCC tumor | Patient 4 | Male | 70-80 | 2-3 |
| RCC-PR4-Normal | Adjacent normal | Patient 4 | Male | 70-80 | / |
| RCC-PR5-PTumor1 | ccRCC tumor | Patient 5 | Male | 50-60 | 2-3 |
| RCC-PR5-PTumor2 | ccRCC tumor | Patient 5 | Male | 50-60 | 2-3 |
| RCC-PR5-PTumor3 | ccRCC tumor | Patient 5 | Male | 50-60 | 2-3 |
| RCC-PR5-Normal | Adjacent normal | Patient 5 | Male | 50-60 | / |
| RCC-PR6-PTumor | ccRCC tumor | Patient 6 | Male | 70-80 | 3 |
| RCC-PR6-Normal | Adjacent normal | Patient 6 | Male | 70-80 | / |
| RCC-BM1-PTumor | ccRCC tumor | Patient 10 | Male | 50-60 | 4 |
| RCC-BM1-Normal | Adjacent normal | Patient 10 | Male | 50-60 | / |

**Table S4.** The nucleic acid sequences used in this study.

| Genes | Primer (5’-3’) |
| --- | --- |
| AXL-F | 5’-GTGGGCAACCCAGGGAATATC-3’ |
| AXL-R | 5’-GTACTGTCCCGTGTCGGAAAG-3’ |
| GAPDH-F | 5’-GAAGGTGAAGGTCGGAGTC-3’ |
| GAPDH-R | 5’-GAAGATGGTGATGGGATTTC-3’ |
| CD163-F | 5’-TTTGTCAACTTGAGTCCCTTCAC-3’ |
| CD163-R | 5’-TCCCGCTACACTTGTTTTCAC-3’ |
| CD206-F | 5’-TCCGGGTGCTGTTCTCCTA-3’ |
| CD206-R | 5’-CCAGTCTGTTTTTGATGGCACT-3’ |
| ARG1-F | 5’-GTGGAAACTTGCATGGACAAC-3’ |
| ARG1-R | 5’-AATCCTGGCACATCGGGAATC-3’ |
| siAXL | 5’-CGTGGAGAACAGCGAGATTTA-3’ |

**Supplementary Figures**

**Figure S1.** Quality control of single-cell data and the distribution of cell composition. (A). Quality control of single-cell RNA-seq data (UMI count, Gene count and Percentage of mitochondrial-related genes). (B). Boxplot compared the distribution of cell composition among major cell types. (C). The distribution of cell composition among major cell types. (D). UMAP plot showed the distribution of different samples. (E). Benchmarking test for single-cell integration.

ns: no significant; *: P < 0.05; **: P < 0.01; ***: P < 0.001.


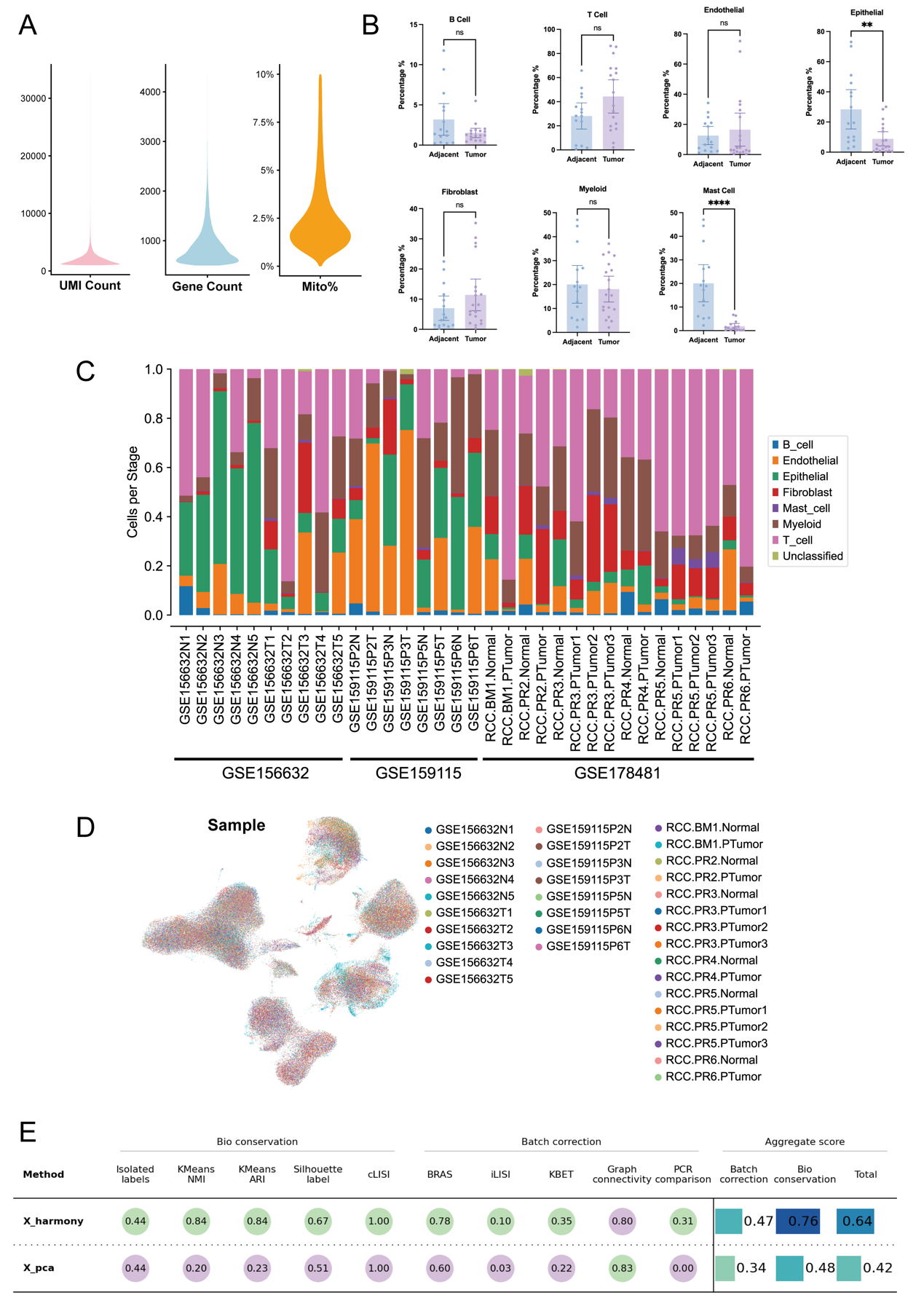


**Figure S2.** Using “SEACells” combined with “DESeq2” pseudo-bulk analysis to identify differentially expressed genes. (A). The SEACell-based Metacells construction results (B). The volcano plot of the differential expression analysis.


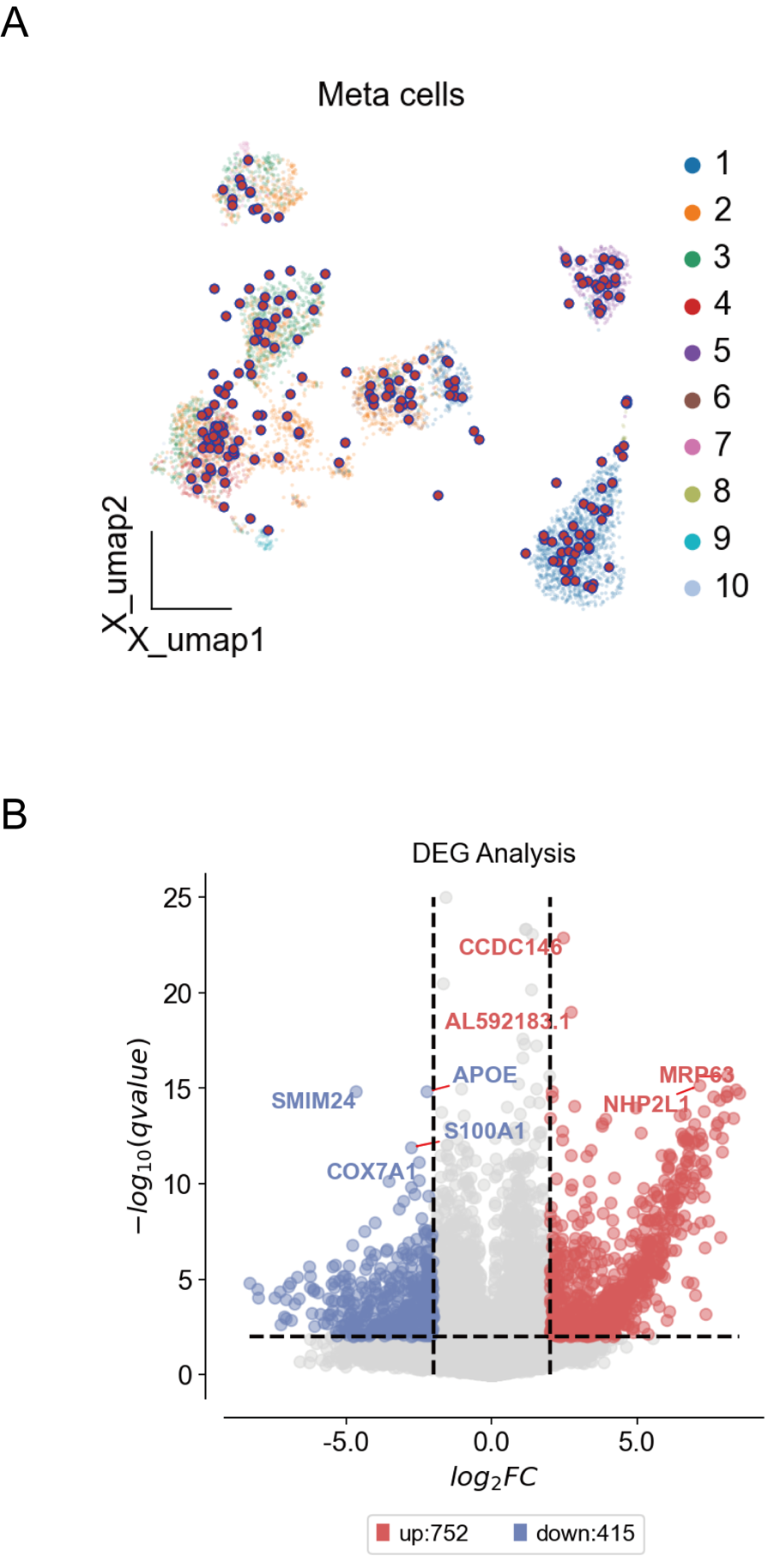


**Figure S3.** Machine learning and SHAP values were used to select essential genes and explain the importance of each gene. (A). SHAP value summary plots demonstrated the importance of each gene. (B). SHAP value force plots aim to illustrate the importance of each gene. (C). The Venn diagram shows the common essential genes identified by the three machine learning algorithms.


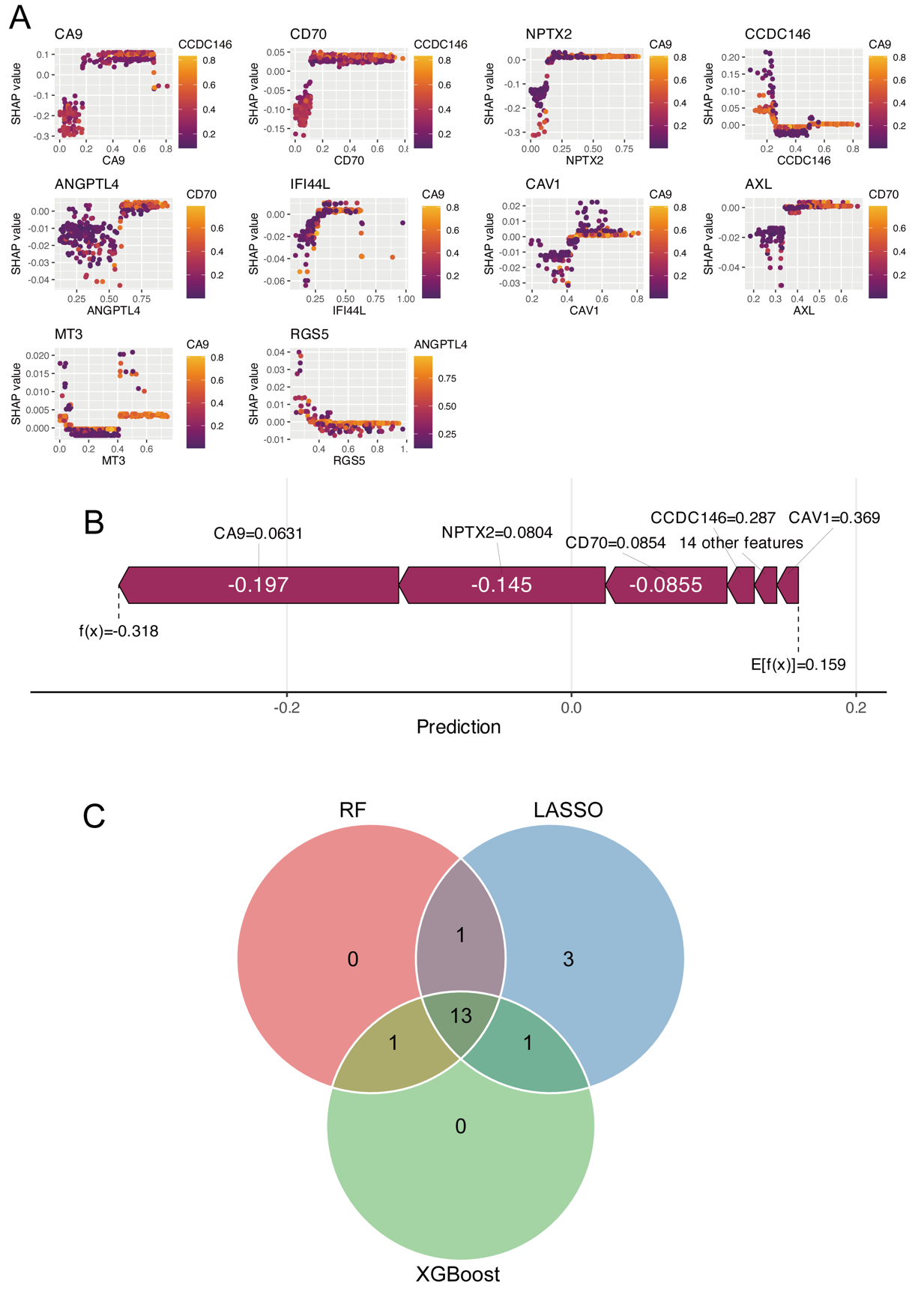


**Figure S4.** The expression level of AXL in TCGA pan-cancer datasets and association with immune cell infiltration among tumor micro-environment. (A). The expression level of AXL in TCGA pan-cancer datasets. (B). The expression level of AXL in TCGA pan-cancer datasets among paired samples. (C). Correlation between AXL and ESTIMATE Scores. (D). Landscapes of immune cell infiltration between high-AXL and low-AXL subgroups using Cibersoft algorithm. (E). Correlation between AXL expression level and M2 macrophage.


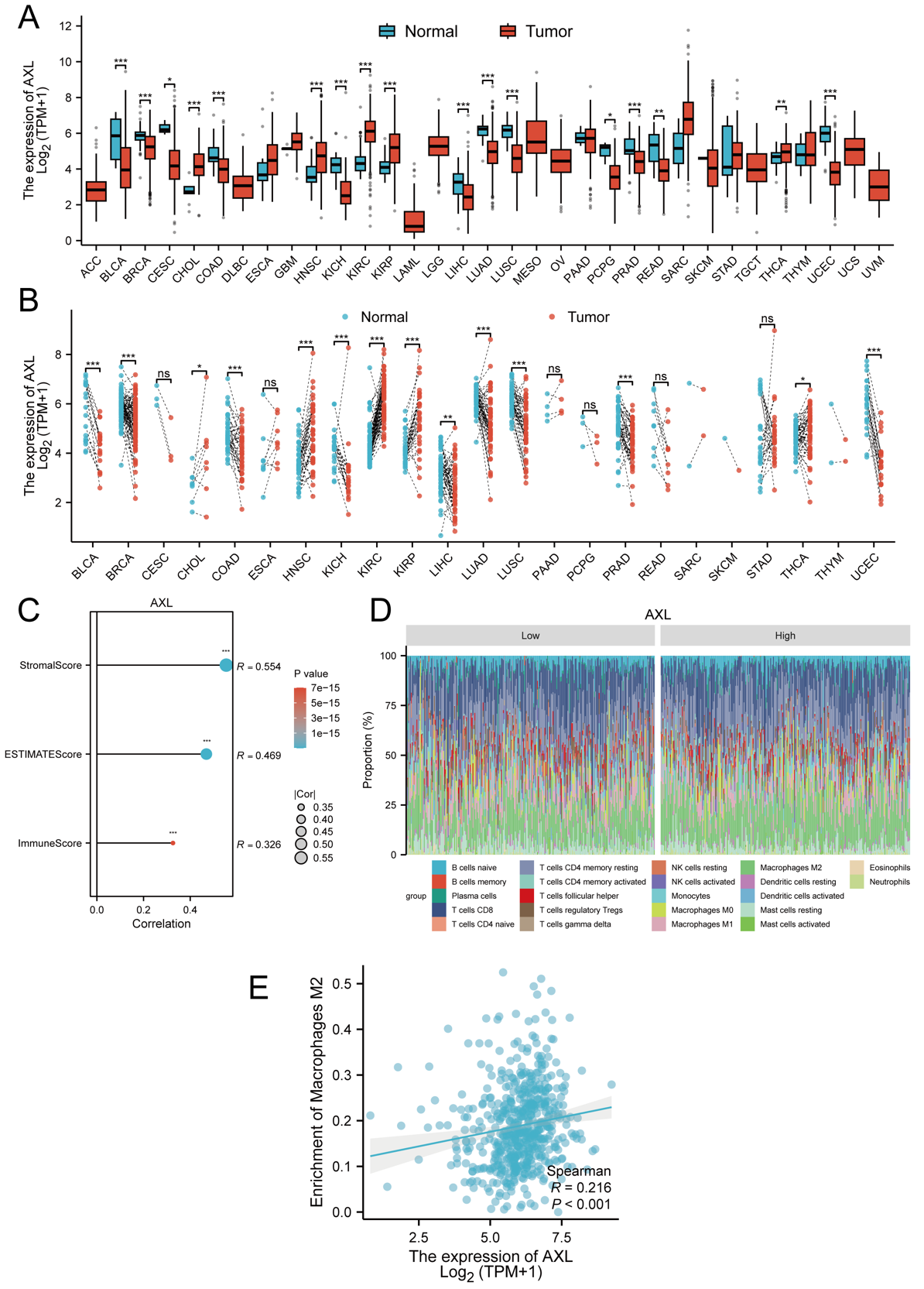


**Figure S5.** Functional enrichment analysis of AXL in TCGA ccRCC dataset. (A). GSEA plots of AXL using Hallmark gene sets. (B). GSEA cluster plots showed AXL involving pathways using KEGG gene sets. (C). GSEA cluster plots showed AXL involving pathways using Reactome gene sets.


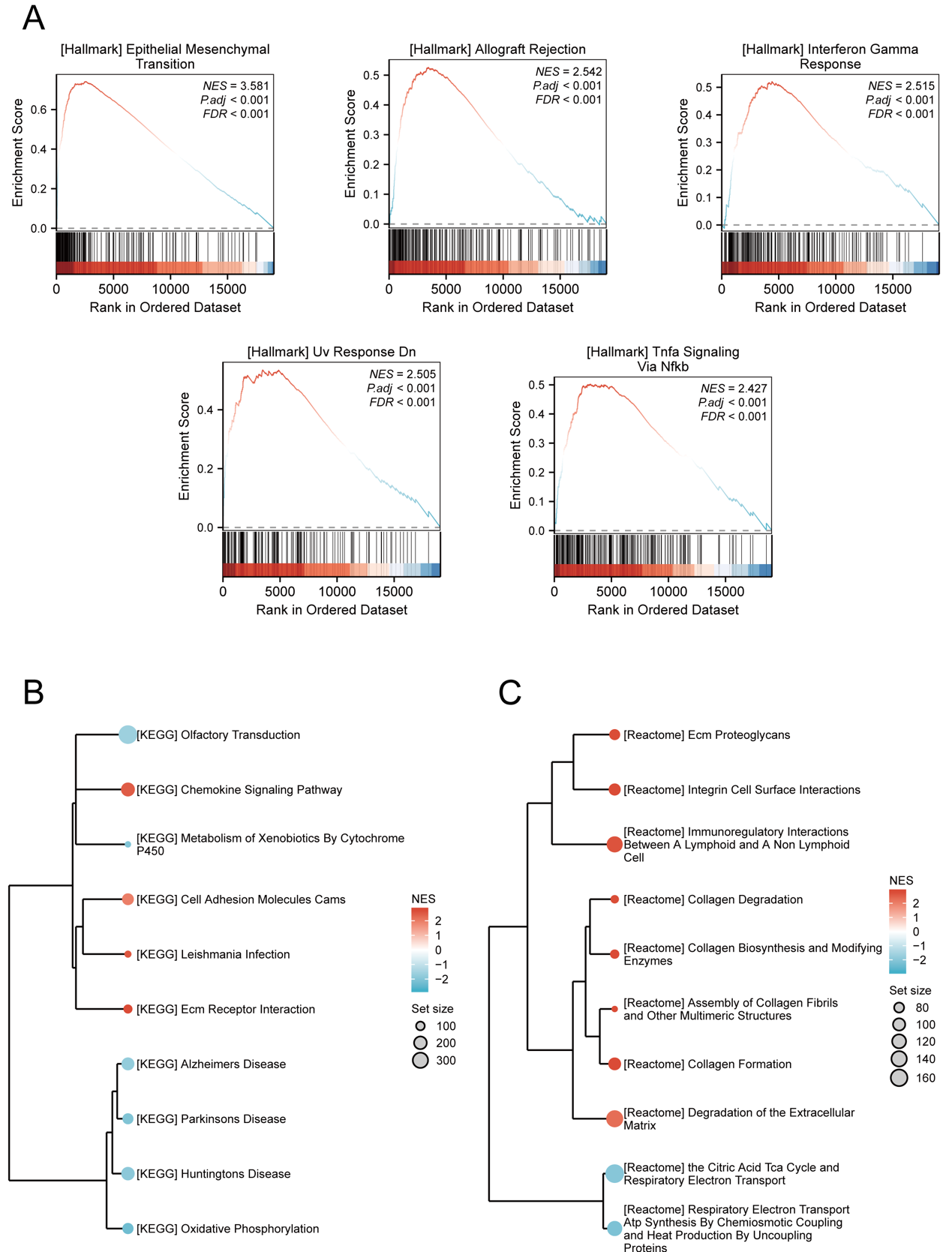


**Figure S6.** Hematoxylin and Eosin (H&E) staining and quality control results of spatial transcriptomics analysis among 5 ccRCC samples.


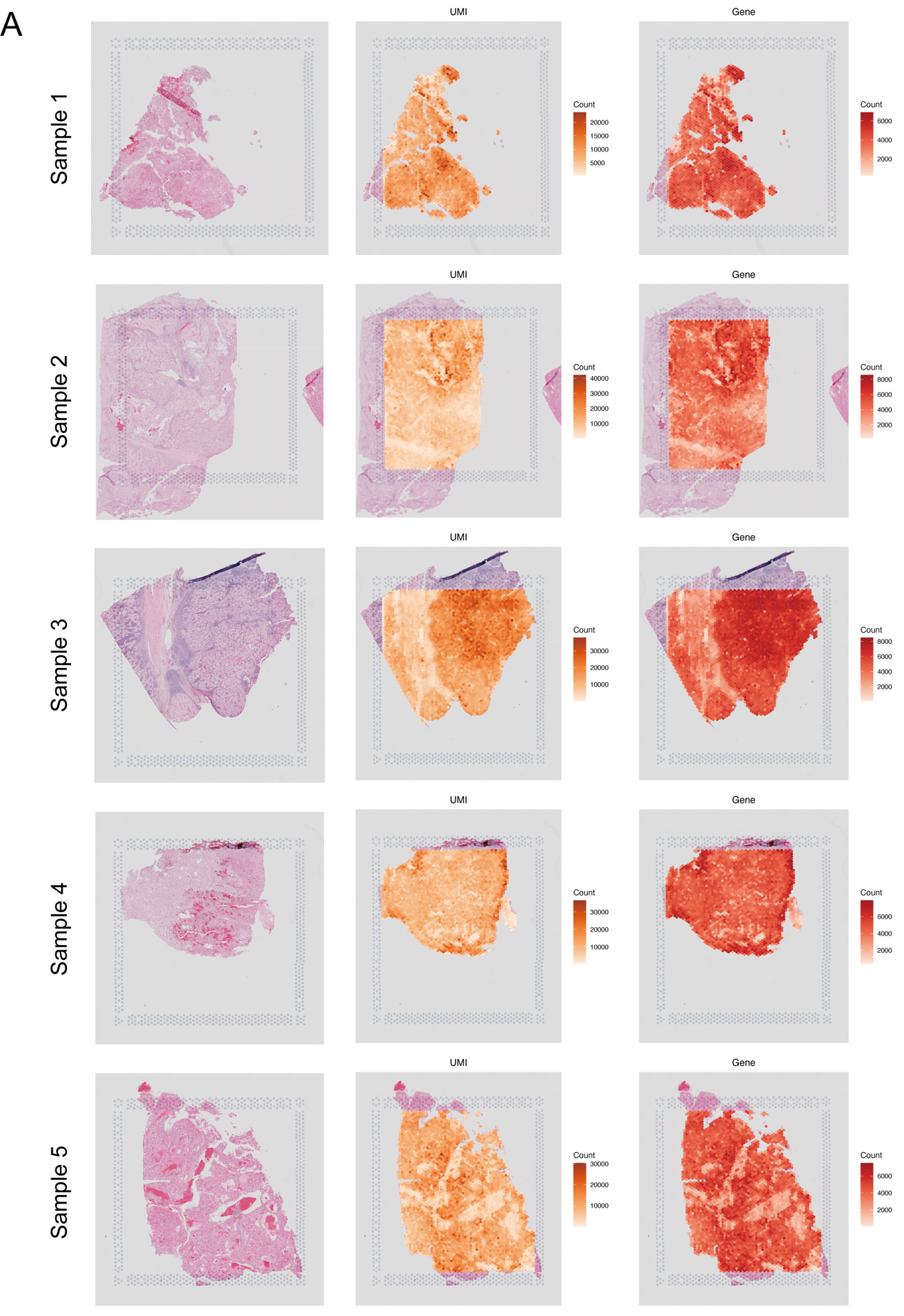


**Figure S7.** Spearman correlation analysis to quantitatively assess spatial co-localization.


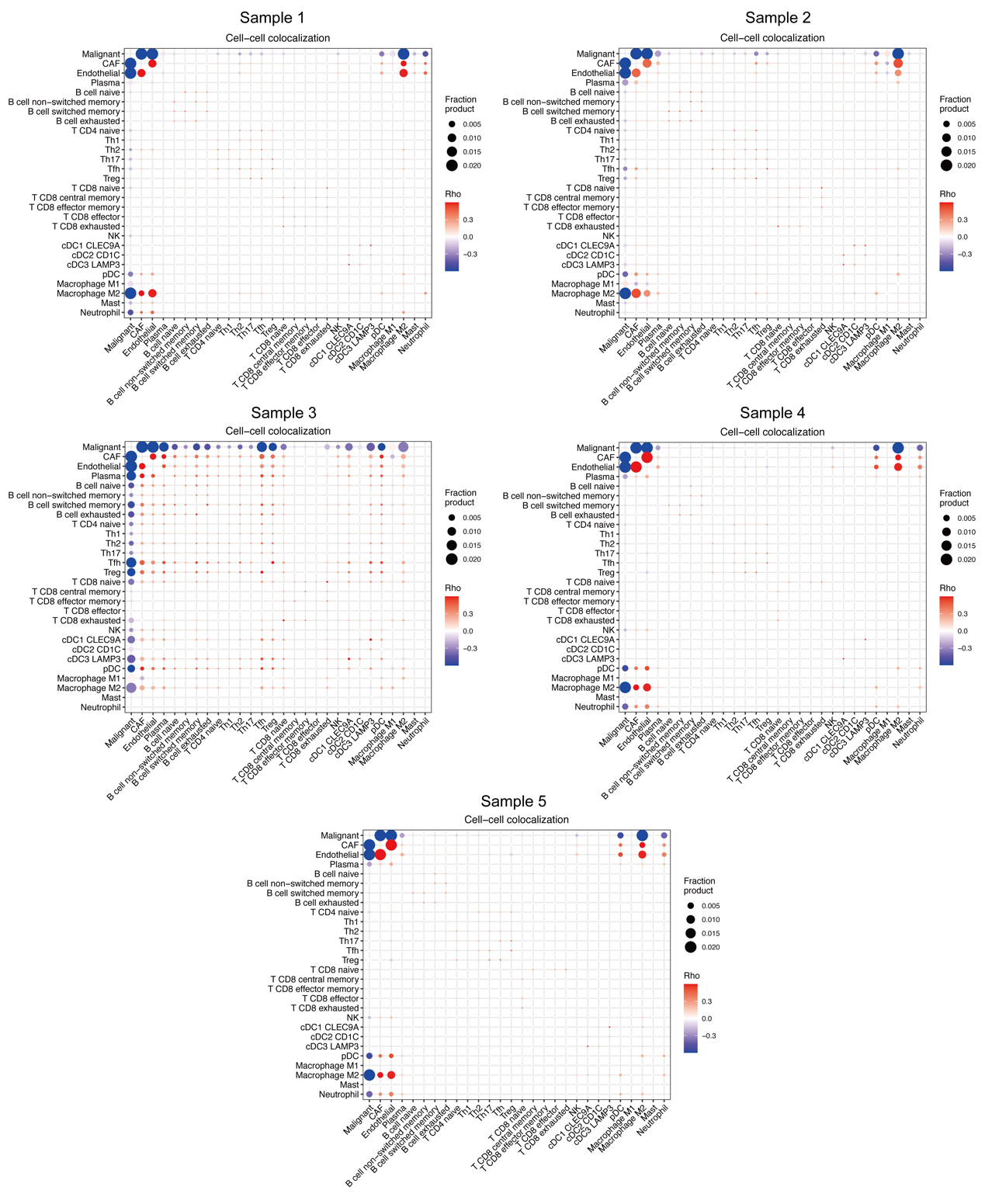

Supplement: Supplementary file 1 — Supplementary Material 1 [file 41065_2025_563_MOESM1_ESM.docx]
